# RNAi-based small molecule repositioning reveals clinically approved urea-based kinase inhibitors as broadly active antivirals

**DOI:** 10.1371/journal.ppat.1007601

**Published:** 2019-03-18

**Authors:** Markus Lesch, Madlen Luckner, Michael Meyer, Friderike Weege, Isabella Gravenstein, Martin Raftery, Christian Sieben, Laura Martin-Sancho, Aki Imai-Matsushima, Robert-William Welke, Rebecca Frise, Wendy Barclay, Günther Schönrich, Andreas Herrmann, Thomas F. Meyer, Alexander Karlas

**Affiliations:** 1 Department of Molecular Biology, Max Planck Institute for Infection Biology, Berlin, Germany; 2 Steinbeis Innovation Center for Systems Biomedicine, Falkensee, Germany; 3 Group of Molecular Biophysics, Department of Biology, Humboldt-Universität zu Berlin, Berlin, Germany; 4 Institute of Virology, Charité University Medicine, Berlin, Germany; 5 Section of Virology, Department of Medicine, Imperial College London, St Mary's Campus, London, United Kingdom; Icahn School of Medicine at Mount Sinai, UNITED STATES

## Abstract

Influenza viruses (IVs) tend to rapidly develop resistance to virus-directed vaccines and common antivirals targeting pathogen determinants, but novel host-directed approaches might preclude resistance development. To identify the most promising cellular targets for a host-directed approach against influenza, we performed a comparative small interfering RNA (siRNA) loss-of-function screen of IV replication in A549 cells. Analysis of four different IV strains including a highly pathogenic avian H5N1 strain, an influenza B virus (IBV) and two human influenza A viruses (IAVs) revealed 133 genes required by all four IV strains. According to gene enrichment analyses, these strain-independent host genes were particularly enriched for nucleocytoplasmic trafficking. In addition, 360 strain-specific genes were identified with distinct patterns of usage for IAVs versus IBV and human versus avian IVs. The strain-independent host genes served to define 43 experimental and otherwise clinically approved drugs, targeting reportedly fourteen of the encoded host factors. Amongst the approved drugs, the urea-based kinase inhibitors (UBKIs) regorafenib and sorafenib exhibited a superior therapeutic window of high IV antiviral activity and low cytotoxicity. Both UBKIs appeared to block a cell signaling pathway involved in IV replication after internalization, yet prior to vRNP uncoating. Interestingly, both compounds were active also against unrelated viruses including cowpox virus (CPXV), hantavirus (HTV), herpes simplex virus 1 (HSV1) and vesicular stomatitis virus (VSV) and showed antiviral efficacy in human primary respiratory cells. An *in vitro* resistance development analysis for regorafenib failed to detect IV resistance development against this drug. Taken together, the otherwise clinically approved UBKIs regorafenib and sorafenib possess high and broad-spectrum antiviral activity along with substantial robustness against resistance development and thus constitute attractive host-directed drug candidates against a range of viral infections including influenza.

## Author summary

Conventional medications against influenza infections, including vaccination and antiviral drug therapy, are targeted against viral determinants–an approach collectively referred to as pathogen-directed. However, influenza viruses mutate fast and quickly develop resistance to these pathogen-directed treatments. An alternative, yet not well established, is to block host cellular molecules required by the virus to successfully multiply. Such a host-directed approach is anticipated to be more robust against the development of drug resistance. This notion is founded on the different modes of action of the two principal approaches: Virus-directed therapeutics target the virus itself. Thus, just a single mutation could abrogate sensitivity to a virus-directed therapeutic. In contrast, it is unlikely that viruses can easily circumvent a pharmacological blockage of a cellular factor by means of just a few mutations. Instead, the virus needs to either exploit an immediate parallel cellular pathway or adjust its replication cycle to a different cellular factor–the latter being a process likely to require multiple mutations, if possible at all. To identify the most promising targets for a host-directed therapy, we performed a small interfering RNA (siRNA) screen with four different influenza virus strains using a lung epithelial cell line. Subsequently, we tested a series of drugs, specific for the products of the genes that are required for replication of all four influenza virus strains tested. Regorafenib and sorafenib, two chemically related urea-based kinase inhibitors already clinically approved for cancer treatment, turned out to be effective inhibitors of all influenza viruses and displayed low cytotoxicity. These drugs blocked viral replication at an early stage of the life cycle not only in cell lines but also in human primary respiratory cells. Moreover, these drugs exhibited high efficacy even against unrelated viruses. In addition, no development of resistance was observed against regorafenib, which was used in an *in vitro* assay representatively of urea-based kinase inhibitors. Our results suggest that regorafenib and sorafenib are promising drug candidates for a host-directed therapy of influenza and other viral infections.

## Introduction

IVs are the cause of seasonal epidemics with up to 500,000 fatalities per year [1] as well as occasional pandemics with increased infection and mortality rates [2]. Epidemics are usually caused by human IAV strains of the subtypes H1N1 and H3N2, as well as IBVs. The source for influenza pandemics has in the past often been provided by avian IAVs that managed to adapt to humans as a host. Current preventive and therapeutic measures target viral proteins and include vaccination as well as direct-acting antivirals (DAAs). Unfortunately, a single mutation of the targeted site can lower the efficacy of a virus-directed drug by two to three orders of magnitude. Consequently, isolates resistant against all approved DAAs have been identified [3–5]. Thus, there is an urgent need for new anti-IV drugs which are less prone to the development of resistance.

IAVs and IBVs encode only 10 to 16 proteins and rely heavily on various host cellular functions to multiply. As cellular proteins are integral parts of the viral life cycle, they also represent medical intervention points. The CCR5-inhibitor maraviroc, which is used in the therapy of human immunodeficiency virus infection, provides the proof-of-concept for host-directed therapy of an infectious disease [6]. The expected advantage of targeting cellular factors over viral factors is that resistance is less likely to develop: even if the virus mutates, the cellular protein will remain blocked. The only way for the virus to develop drug resistance is to utilize alternative cellular pathways. Such alternatives exist for certain pathways, highlighted by the ability of IVs to adapt to different types of sialic acids as receptors [5] or different importins for nuclear import of viral ribonucleoprotein particles (vRNPs) [7, 8]. However, there might also be cellular bottlenecks to which development of resistance is impeded. One example for a drug targeting such a bottleneck is the microRNA-122 inhibitor miravirsen, which underwent clinical trials for treatment of hepatitis C virus infection [9], and for which a very high barrier against development of resistance was observed [10]. To systematically identify the human genes exploited by IVs, several loss-of-function screens have been conducted by independent laboratories including our own [11–13]. In these studies, more than 1,000 genes have been proposed to be growth relevant. However, mainly IAVs of subtype H1N1, such as A/WSN/1933(H1N1) (WSN) and A/Puerto Rico/8/1934(H1N1), have been used in the published screens. Thus, it is unclear to which extent the published genes are relevant for other IV types, such as H3N2 IAV, avian-adapted IAVs, or IBVs, and which genes are best suited for a pan-specific host-directed therapy of influenza.

Here, we performed a large-scale, comparative small interfering RNA (siRNA) screen with a human H1N1 IAV (WSN), a human H3N2 IAV (A/Panama/2007/1999(H3N2), PAN), an avian H5N1 IAV (A/Vietnam/1203/2004(H5N1), VN), and an IBV (B/Thuringia/02/2006xB/Vienna/33/2006, THW) to identify suitable targets for a host-directed influenza therapy.

Next, we screened drugs targeting genes required for replication of all four viruses for antiviral activity. Costs for development of a new drug are tremendously high. A more cost-efficient way to generate drugs for a new indication is to repurpose existing drugs approved for other indications. Successful examples of repurposed drugs include bupropion, chlorpromazine, and celecoxib [14]. We identified the UBKIs regorafenib [15] and sorafenib [16]–approved for specific cancer indications–as highly promising candidates for a host-directed therapy of influenza.

## Results

### Comparative siRNA screen identifies IV strain-independent host factors

**Fig 1:**
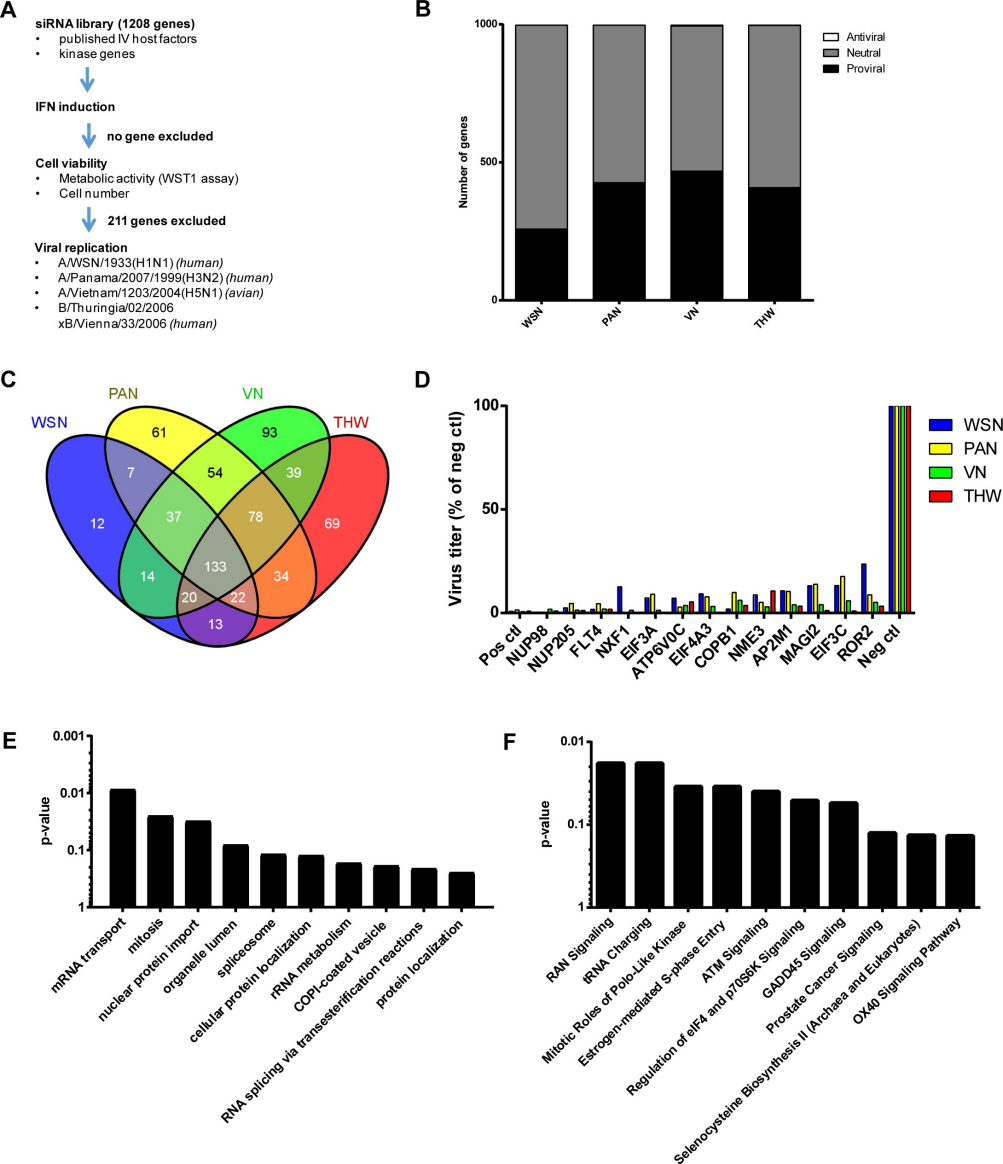
Result of the siRNA screen. (A) Setup of the siRNA screening campaign. (B) Classification of the 997 genes investigated with the four IV strains. (C) Venn diagram of genes required for replication of the individual viruses. (D) Virus titers relative to negative control upon knockdown of genes most strictly required for IV replication. Data represent the mean of all siRNAs targeting the specified gene. Data are from the siRNA screen. Positive control: IAV segment 5 (WSN, PAN, VN), XPO1 (THW). Negative control: Non-targeting scrambled sequence siRNA. (E) The genes required for replication of all four IV strains were subjected to gene group enrichment analysis using DAVID functional annotation clustering. Data represent the geometric means of the clusters’ p‐values for the top 10 ranking clusters. (F) The same data as in (E) were analyzed using IPA for enrichment of canonical pathways. Data represent the pathways’ p‐values for the top 10 ranking pathways.

The siRNA screen was set up as shown in Fig 1A. Based on previous loss-of-function studies with IVs, we designed an siRNA library targeting 128 genes identified to be growth relevant in at least two out of a panel of six published screens [12], the 461 genes identified in the primary screen of our own group [11], and 717 genes associated with the human kinome, since kinase genes were the most overrepresented gene category in several loss-of-function-screens [17]. In total, this library targeted 1,208 genes with 3,482 siRNAs (Supplementary S1 Table). We first screened the library for immunogenicity and cytotoxicity to exclude siRNAs which induce an interferon (IFN) response and cause cell death, respectively. We identified 678 cytotoxic siRNAs, but no immunogenic siRNAs. In total, we excluded 211 genes from further analyses due to the cytotoxicity of the corresponding siRNAs, leaving 997 genes. Subsequently, the library was screened for production of infectious virus progeny. First, the raw data of the virus replication screens were normalized to a non-targeting siRNA and an siRNA targeting a proviral gene (IAV: viral NP gene, IBV: cellular gene XPO1). Then, the normalized data for all siRNAs targeting a given gene were summarized by the collective mean and the collective strictly standardized mean difference (cSSMD), as described previously [18]. Employing collective mean > 0.5 and cSSMD ≥ 1 as cut-off criteria, we identified between 258 (WSN) and 468 (VN) proviral genes for each virus (Fig 1B). A set of 133 genes was identified as proviral in all four virus replication screens (strain-independent genes) (Fig 1C). Based on the average effect of the siRNAs tested, knockdown of thirteen genes impaired viral replication by an order of magnitude or more (Fig 1D). We analyzed these strain-independent genes for enrichment of gene groups using the Database for Annotation, Visualization and Integrated Discovery (DAVID) [19] (Fig 1E) and/or Ingenuity Pathway Analysis (IPA, Ingenuity H Systems; http://www.ingenuity.com) (Fig 1F). We combined the results depicted in Fig 1D–1F and visualized them as interaction map (S1 Fig). Taking these results together, the nucleocytoplasmic transport appears to be the cellular process which is most sensitive to perturbation with respect to replication across all IV strains tested. Consequently, nucleocytoplasmic trafficking likely represents an attractive target for the development of host-directed therapeutics. Further highly sensitive processes include cell cycle regulation, RNA splicing, translation, and tRNA charging. Interestingly, the latter has not been identified as enriched in previous studies. The full results of the screens on siRNA and gene level, as well as the gene group enrichment analyses, are shown in Supplementary S2–S5 Tables. Of note, we did not identify any factor which was antiviral for all four strains, probably because our gene set was selected to identify proviral genes.

### Comparative siRNA screen identifies strain-specific host-factors and cellular processes

**Fig 2:**
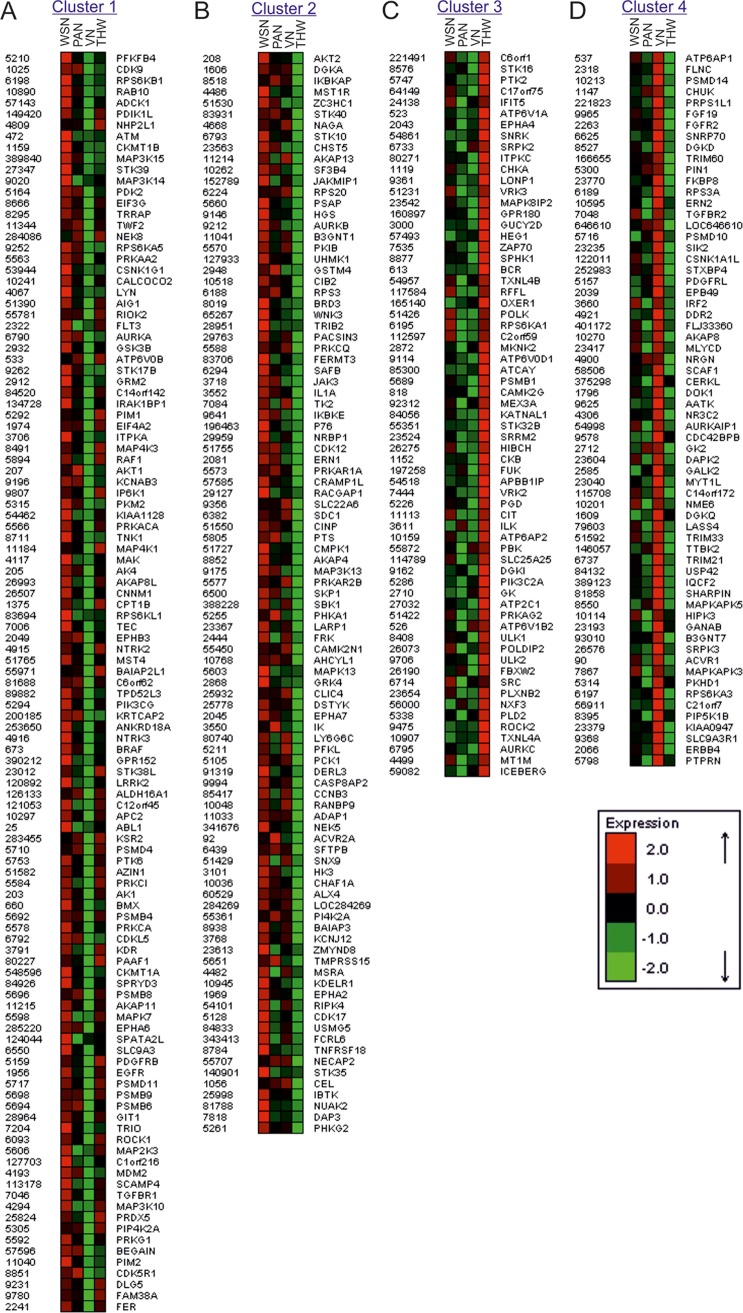
Several genes are strain-specifically required. Strain-specific genes were identified by mixed effects analysis and clustered using the CLICK algorithm [79]. Data represent the mean of the normalized viral load for the siRNAs targeting the individual genes. Data analyzed are from the screen outlined in Fig 1A.

Although the primary aim of this study was to identify genes universally required by all prototypes of IVs, we also searched for entities differentially required between the four viral strains. We therefore analyzed the data sets with a hierarchical statistical model based on linear mixed effects. Subsequently, we filtered the results and obtained 360 strain-specific genes. By clustering these genes we found four clusters (S2A Fig). Genes in cluster 1 were especially relevant for the avian IAV VN but much less for the human H1N1 IAV WSN (Fig 2A). The genes in cluster 2 were especially relevant for the IBV strain THW and more or less dispensable for WSN (Fig 2B). Genes in cluster 3 were especially relevant for VN and the human H3N2 IAV PAN but much less for THW (Fig 2C). Genes in cluster 4 were most essential to replication of THW but much less for VN (Fig 2D). Twenty-one genes could not be assigned to any cluster (S2B Fig). Taking the results from the cluster analysis of the strain-specific genes together, most of the differences were identified between the IAVs and the IBV, or the human IVs and the avian IV.

We extended our search from differentially required genes to differentially required pathways, cellular compartments, and molecular functions. For this, we first queried the Gene Ontology database [20], the Kyoto Encyclopedia of Genes and Genomes database [21], and the Reactome database [22] for terms represented by ≥ 2 tested genes and again identified terms with strain-specific effects by a mixed effects model. Overall, 3,235 terms were represented by our siRNA library and 884 of these displayed significantly different effects on the four viral strains. Two processes for which the avian IV strain VN was less sensitive than the three IAVs were nuclear factor kappa-light-chain-enhancer of activated B cells (NF-кB) activation (S3A Fig and S6 Table) and regulation of RNA splicing (S3B Fig and S7 Table). Another cellular function with strain-dependent relevance is the cellular RNA polymerase activity, for which interference affected the IBV strain the most and the avian IV the least (S3C Fig and S8 Table). In summary, we often observed strain-specific host cell genes or processes to differ between IAVs and the IBV, or between human IVs and the avian IV. The full list of differentially required genes and gene ontologies and pathways can be found in S9 and S10 Tables.

### Small molecule screen identifies the urea-based kinase inhibitors regorafenib and sorafenib as drugs suitable for repurposing for influenza treatment

**Fig 3:**
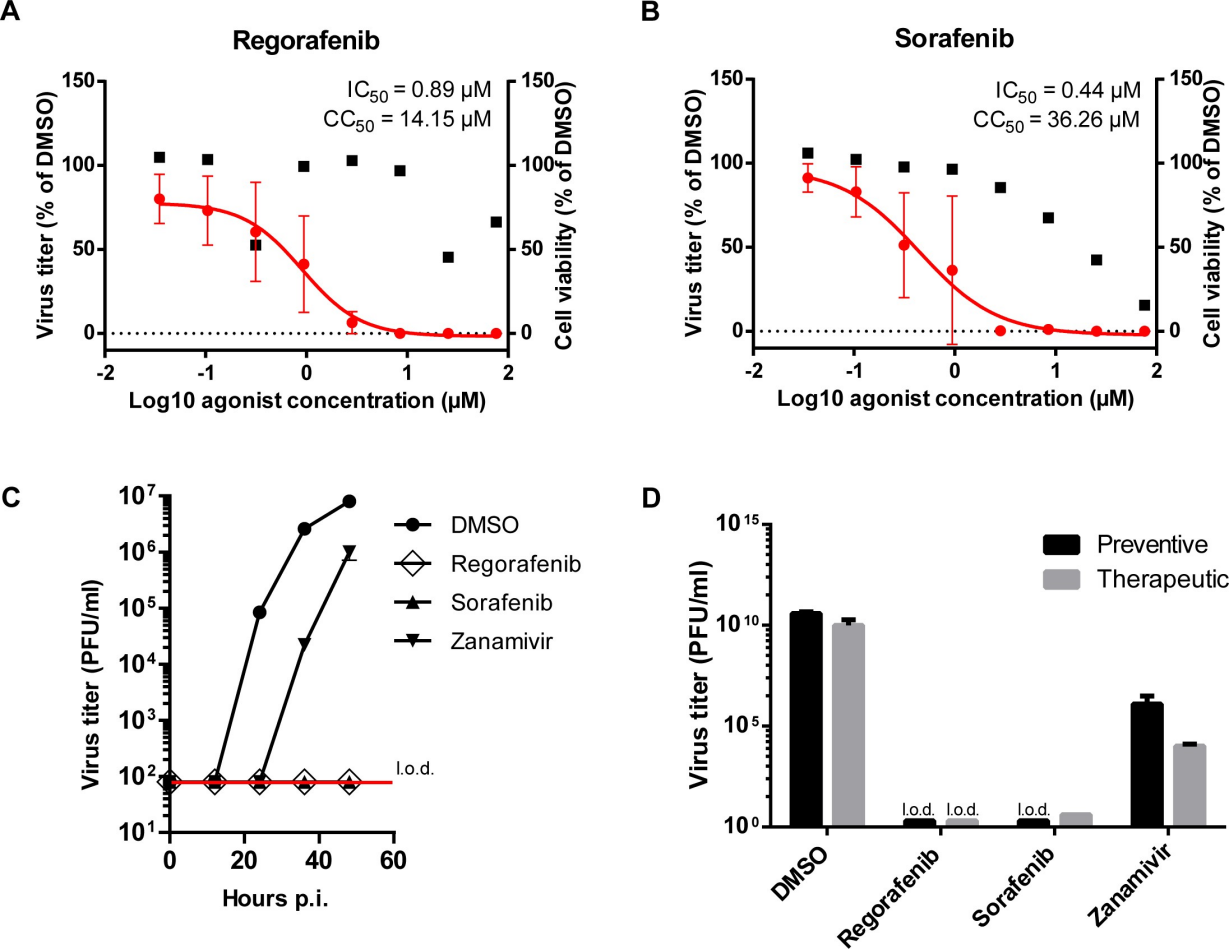
Several clinically approved and experimental drugs block multiple round and primary infection of IV. (A & B) For determination of virus replication, A549 cells were pre-treated with small molecules at different concentrations for 2 h. Cells were infected with IV strain WSN and cultivated for 36 h in presence of small molecules. Finally, virus titers in supernatants were determined and the infection rate was selected as read-out. IC_50_: half maximal inhibitory concentration of drug in virus replication assay. For determination of cell viability, A549 cells were cultivated for 36 h in presence of small molecules at different concentrations prior to conduction of WST-1 assay. Data represent signal in WST-1 assay relative to the vehicle control expressed as mean ± SEM of n = 3 technical replicates. CC_50_: half maximal inhibitory concentration in WST-1 assay. (A) Regorafenib. (B) Sorafenib. (C) A549 cells were pre-treated with small molecules at non-toxic concentrations (regorafenib & sorafenib, 3 μM; zanamivir, 1 μM) for 2 h. Cells were infected and cultivated for 48 h in presence of small molecules. Virus load was assessed in 12-hour intervals by plaque assay. Data represent mean ± SD of technical replicates. l.o.d.: limit of detection. Log-transformed virus titer for zanamivir treatment was compared to control using multiple linear regression. The titer for DMSO exceeds the one for zanamivir treatment significantly by a factor of 10^4^ (p < 0.001; 95%CI: 10^2^ to 10^6^). (D) A549 cells were treated with small molecules (same doses as in C) either starting 2 h prior to infection (preventive, black bars) or 4 h post-infection (therapeutic, grey bars) with WSN. Infected cells were cultivated for 36 h in presence of small molecules and virus load was assessed by plaque assay. Data represent mean ± SD of technical replicates. Log-transformed virus titer data were analysed with a linear model. The titer for preventive treatment exceeds the titer for therapeutic treatment (p = 0.041). It was lower for zanamivir treatment compared to DMSO (p ≤ 0.001). For preventive treatment with sorafenib and for both treatments with regorafenib the titer was below the limit of detection (l.o.d). Please note that zanamivir was applied at suboptimal concentration in (C) and (D).

To identify drugs suitable for repositioning towards a host-directed anti-influenza therapy, we screened the 133 strain-independent pro-IV genes for approved drugs or drugs in clinical trials, using the Ingenuity database, identifying 41 drugs targeting 14 genes (Table 1). We screened 39 of these drugs and four related compounds for antiviral activity and cytotoxicity in A549 cells. Seventeen drugs inhibited multiple round replication of WSN with an IC_50_ < 10 μM (Supplementary S11 Table). For fourteen of these highly active compounds, the selectivity index (SI) was > 10, which we considered a useful therapeutic window. Three of these drugs targeted the gene FLT4, which was also among the top three hits in the siRNA screen (Fig 1D). Therefore, we decided to focus on two of the FLT4 inhibitors—regorafenib and sorafenib—in more detail. We determined the SIs of regorafenib and sorafenib to be 16 and 82, respectively (Fig 3A and 3B), when adding the agents 2 h prior to infection. After quantifying the dose dependency of the antiviral effect of regorafenib and sorafenib, we investigated the time dependency of both compounds, employing the highest non-cytotoxic dose (3 μM each). While the virus titer in the supernatant of DMSO-treated cells increased up to 8 x 10^6^ PFU/ml until 48 h post infection (p.i.), we could not detect virus in the supernatant of regorafenib- or sorafenib-treated cells (Fig 3C). Subsequently, we compared preventive (2 h prior to infection) and therapeutic (4 h post infection) application of both drugs (Fig 3D). Using the preventive approach, no virus could be detected with either of the two compounds. When applying the drugs therapeutically, a more than 10^9^-fold reduction of the viral load was observed with sorafenib, and no virus could be detected in the supernatant of regorafenib-treated cells.

**Table 1:** Druggable genes required for IV replication.

GeneID	GeneSymbol	Virus titer upon knockdown^§^ (% of neg ctl)	Inhibitory drugs
2324	FLT4	2	CEP 7055, motesanib, telatinib, tivozanib, cabozantinib^†^, axitinib^†^, sorafenib^†^, regorafenib^†^, sunitinib^†^, pazopanib^†^^*a*^, vandetanib^†^
10188	TNK2	12	vemurafenib^†^^*a*^
983	CDK1	15	alvocidib^b^
2475	MTOR	22	dactolisib^*a*^, OSI-027, ridaforolimus, tacrolimus^†^, everolimus^†^, sirolimus^†^, temsirolimus^†^
1017	CDK2	26	BMS-387032, alvocidib^b^
5293	PIK3CD	27	SF 1126, PX-866, dactolisib^*a*^, pictrelisib, buparlisib, XL147, idelalisib
2268	FGR	27	vemurafenib^†^^*a*^
6300	MAPK12	30	talmapimod
64805	P2RY12	31	ticagrelor^†^, prasugrel^†^, clopidogrel^†^, ticlopidine^†^
1586	CYP17A1	33	abiraterone, ketoconazole^†^, abiraterone acetate^†^
3716	JAK1	34	tofacitinib^†^, ruxolitinib^†^
3932	LCK	35	dasatinib^†^, pazopanib^†^^*a*^
1018	CDK3	37	alvocidib^b^
5605	MAP2K2	38	selumetinib, trametinib^†^

^§^data are derived from siRNA screen,

^†^drug is clinically approved in at least one country,

^*a*^drug targets two genes listed in the table,

^*b*^drug targets three genes listed in the table

Taken together, we identified two inhibitors targeting the cellular gene FLT4, which possess high antiviral activity at non-cytotoxic concentrations in A549 cells. Therefore, we decided to characterize the mode of action of FLT4 inhibitors regorafenib and sorafenib in more detail.

### Urea-based kinase inhibitors regorafenib and sorafenib do not affect influenza virus entry

The gene FLT4 encodes the protein vascular endothelial growth factor receptor 3 (VEGFR3), a receptor tyrosine kinase (RTK). Both regorafenib and sorafenib belong to the class of urea-based kinase inhibitors (UBKIs), which are known to inhibit several other kinases as well as VEGFR3 [15, 16, 23]. Consequently, using the CRISPR/Cas9 technology, we generated knockout cell lines for the main targets of regorafenib/sorafenib and examined the effect on replication of the IV strain WSN. The strongest effect was observed with knockdown of FLT4, but silencing of FGFR1, FLT1 (encoding VEGFR1), and PDGFRB also markedly reduced viral replication (S4 Fig). These observations indicate that regorafenib and sorafenib impair viral replication by blocking multiple relevant kinases in parallel–not solely by affecting FLT4.

**Fig 4:**
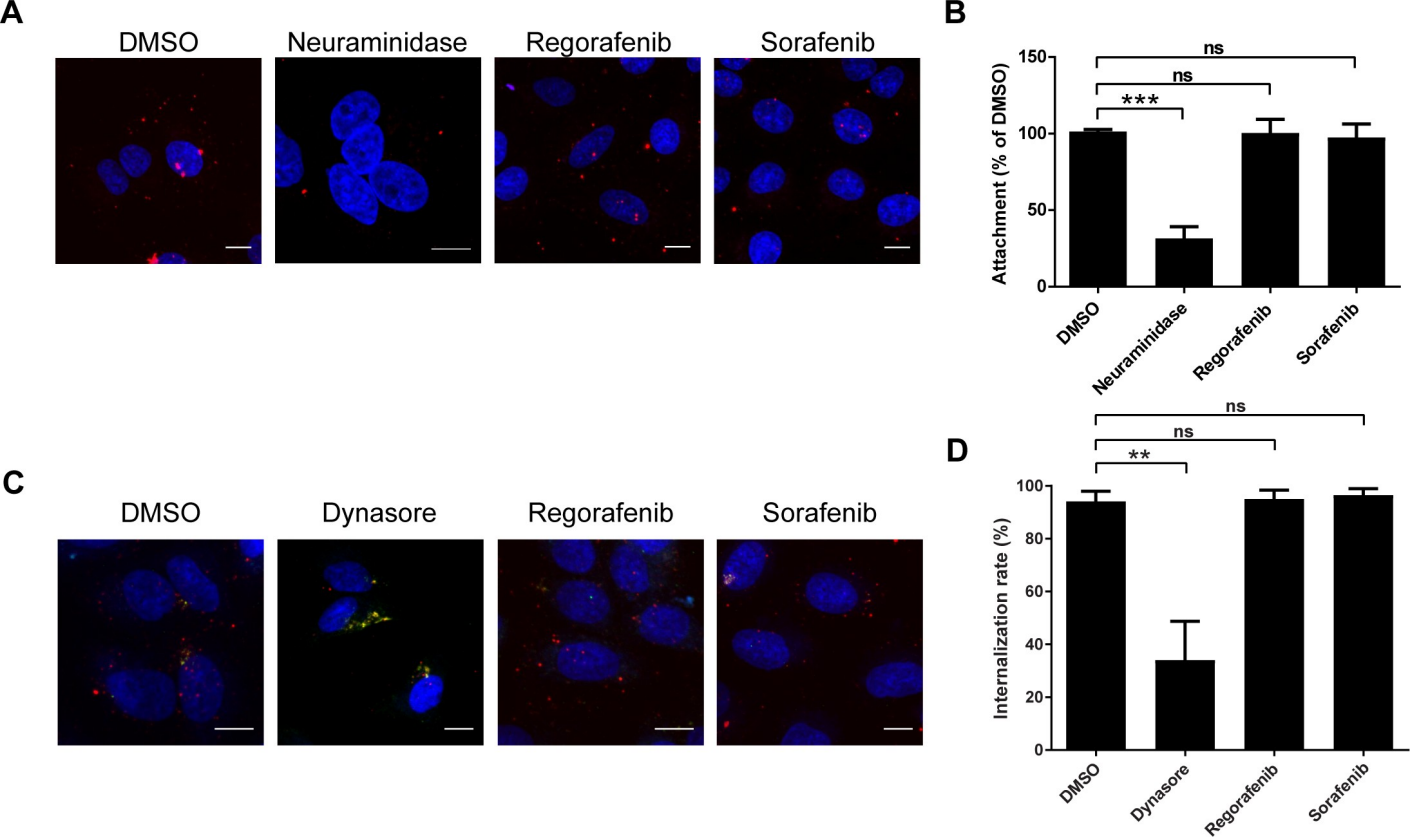
UBKIs do not affect entry of IAV into host cells. (A and B) A549 cells were pre-treated with small molecules at 3 μM, an equivalent amount of DMSO, or *Cp* neuraminidase, for 30 min. Cells were incubated at 4°C with IAV strain PAN labeled with Alexa Fluor 647 (red), fixed and stained with DAPI (blue), and subjected to (A) microscopic analysis (scale bar = 10 μm) or (B) flow cytometry. Data in (B) represent DMSO-normalized mean ± SEM of n = 3 independent experiments (mean values are significantly different: one-way ANOVA: p = 0.0004; Dunnett´s multiple comparison test for comparison with DMSO control, ns: not significant (p-value > 0.05), ***: p-value ≤ 0.001). (C and D) A549 cells were pre-treated with small molecules at 3 μM, an equivalent amount of DMSO, or 100 μM dynasore for 30 min. After virus attachment for 1 h at 4°C, cells were cultivated in presence of the compounds for 45 min at 37°C to induce virus internalization, then stained for DNA (DAPI, blue) and (extracellular) IV hemagglutinin (HA) protein (green) and subjected to (C) microscopic analysis (scale bar = 10 μm) or (D) flow cytometry. Red signals represent internalized virus particles, yellow (sufficiently Alexa Fluor 647-labeled) and green (insufficiently Alexa Fluor 647-labeled) signals represent cell surface-bound virus particles. Data in (D) represent mean ± SEM of n = 3 independent experiments (mean values are significantly different: one-way ANOVA: p = 0.0011; Dunnett´s multiple comparison test for comparison with DMSO control, ns: not significant (p-value > 0.05), **: p-value ≤0.01.

We wanted to identify the main step of the viral replication cycle that is blocked by regorafenib and sorafenib. The IV replication cycle starts with attachment of the virion by binding to sialic acid residues on the host cell surface [24]. Neither regorafenib nor sorafenib affected attachment, whereas *Clostridium perfringens* (*Cp*) neuraminidase, an enzyme removing sialic acid residues from the cell surface, impaired this process considerably, as indicated by fluorescence microscopy (Fig 4A) and flow cytometry (Fig 4B). Upon attachment, IV particles enter the cells mainly via clathrin-mediated endocytosis (CME) [25], although macropinocytosis as an alternative entry route has been described [26]. First, we assessed the effect of regorafenib and sorafenib on the uptake of two conventional substrates of CME: epidermal growth factor (EGF) and transferrin [27, 28]. Neither of the two agents affected the internalization of EGF (S5A Fig) or transferrin (S5B Fig). Accordingly, the internalization of virus particles was not affected by regorafenib or sorafenib treatment, as indicated by fluorescence microscopy (Fig 4C) and flow cytometry (Fig 4D). Thus, we conclude that regorafenib and sorafenib do not affect the internalization of IV by host cells.

### Urea-based kinase inhibitors regorafenib and sorafenib inhibit influenza virus replication by interfering with fusion

**Fig 5:**
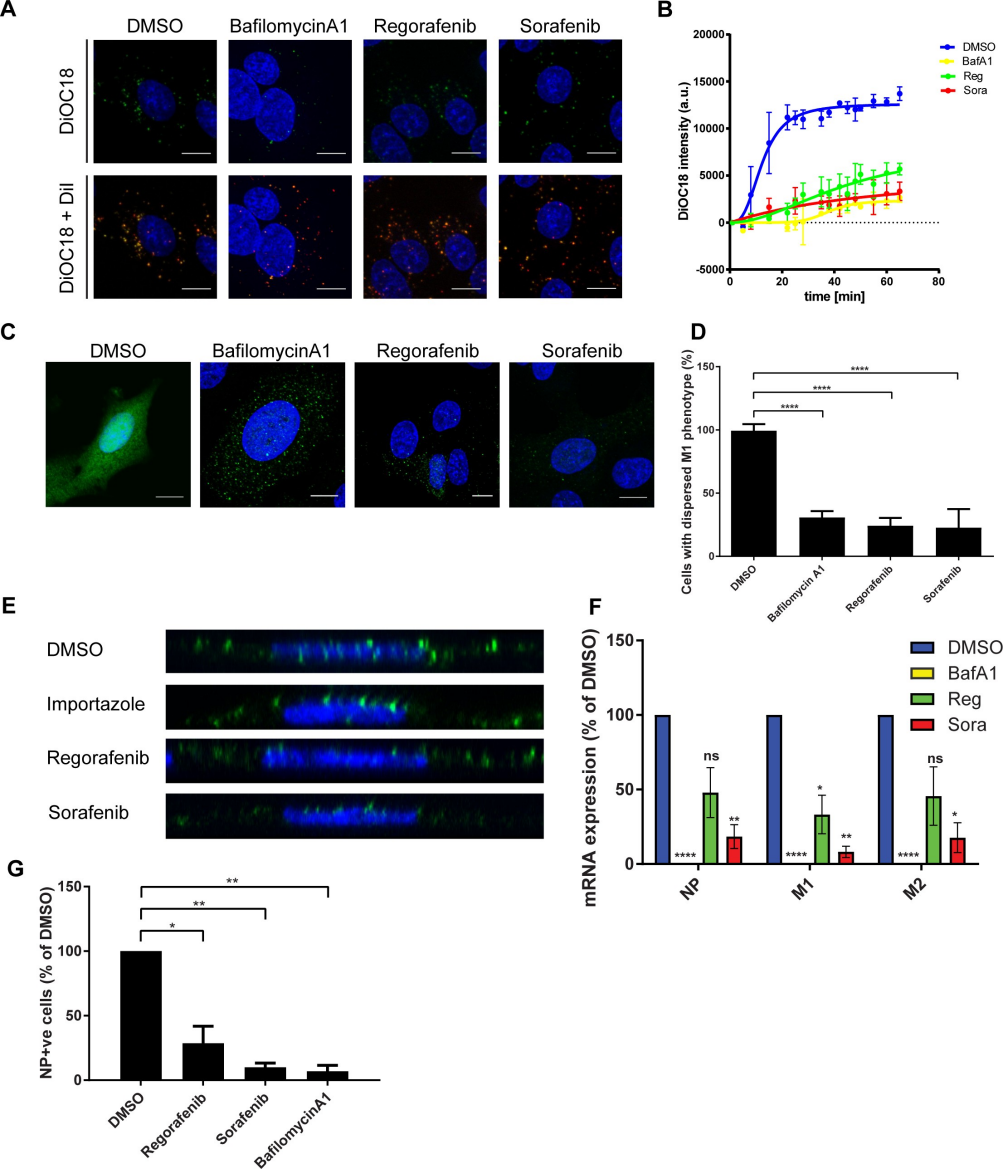
UBKIs block cellular pathways important for fusion and downstream processes. (A and B) A549 cells were pre-treated with Hoechst to stain the nuclei (blue) and either small molecules (regorafenib and sorafenib: 3 μM, bafilomycin A1: 200 nM) or an equivalent amount of DMSO for 30 min before incubation at 4°C with IAV strain PAN labeled with DiOC18 (green) and DiI (red). Virus-containing medium was exchanged against media containing either small molecules or DMSO only, and cells imaged at 37°C for 65 min. In (A), representative images for the DiOC18 and the merge of the DiOC18 and the DiI signal acquired 60 min post infection are shown. Scale bar = 10 μm. In (B), micrographs were quantitatively analyzed and the change of the DiOC18 signal intensity is shown for individual time points. Data represent mean (n = 3) ± SEM of independent experiments. (C and D) A549 cells were pre-treated as described for A and B, incubated at 4°C with IAV strain PAN (MOI 5) and then cultivated for 3 h at 37°C in the presence of small molecules and cycloheximide before staining for DNA with DAPI (blue) and IAV M1 protein (green), and subjected to microscopic and flow cytometry analysis. (C) Representative images of three independent experiments. (D) Quantitative analysis. Data represent mean ± SEM of n = 3 independent experiments (mean values are significantly different: one-way ANOVA: p ≤ 0.0001; Dunnett´s multiple comparison test for comparison with DMSO control: **** indicates p_adjusted_ = 0.0001). (E) A549 cells, pre-treated as described for (A and B), incubated at 4°C with IAV strain PAN (MOI 5) and cultivated for 4.5 h at 37°C in the presence of small molecules and cycloheximide before staining for DNA with DAPI (blue) and IAV NP protein (green), and subjected to microscopic analysis. Representative micrographs of the z-axis of individual cells are shown. (F) A549 cells were pre-treated with small molecules at 3 μM or an equivalent amount of DMSO for 2 h. Subsequently, the cells were infected with IAV strain WSN and cultivated for 4 h in presence of small molecules. RNA was extracted and the relative amount of the NP, M1, and M2 mRNAs determined by qRT-PCR. Data represent mean ± SEM of n = 3 independent experiments. Significance was calculated based on the one sample t-test for comparison with DMSO control: *: p-value ≤ 0.05, **: p-value ≤ 0.01, ****: p-value ≤ 0.0001. (G) A549 cells were pre-treated with small molecules at non-toxic concentrations (regorafenib & sorafenib: 3 μM, bafilomycinA1: 1 μM) for 2 h. Cells were infected and cultivated for 6 h in presence of small molecules before staining for viral nucleoprotein (NP). Data represent mean ± SEM of n = 3 independent experiments of the fraction of NP-positive cells relative to the vehicle control. Significance was calculated based on the one sample t-test for comparison with DMSO control: *: p-value ≤ 0.05, **: p-value ≤ 0.01.

One of the first intracellular steps of the viral replication cycle is the fusion of the viral membrane with the endosomal membrane, a process that depends on endosomal acidification [24]. Again, we first tested the effect of regorafenib and sorafenib on the fate of the classical CME cargos EGF and transferrin, which also depends on endosomal acidification [27, 28]. EGF is typically degraded in lysosomes, whereas transferrin is recycled to the plasma membrane. As expected, the intracellular level of EGF (S6A Fig) and transferrin (S6B Fig) gradually decreased over time in DMSO-treated cells. In contrast, treatment with regorafenib or sorafenib blocked the decrease in intracellular EGF and transferrin levels, most likely due to an impairment of endosomal acidification. Next, we directly characterized the effect of regorafenib and sorafenib on fusion of virus particles with the endosomal membrane. To this end, we simultaneously labeled IV particles with two lipophilic dyes generating a Förster resonance energy transfer (FRET) pair, DiOC18 (green) and Dil (red). Intact labeled virions appear red due to FRET from DiOC18 to DiI, whereas a shift towards green color occurs when the membranes fuse and the fluorophores become dispersed, reducing the FRET [29]. Thus, our data suggest that both regorafenib and sorafenib impair cellular processes relevant for the fusion process, as indicated by qualitative (Fig 5A) and quantitative (Fig 5B) fluorescence microscopic analysis. In line with these findings, the processes downstream of fusion, i.e. the uncoating of vRNPs, as indicated by M1 dispersal [30] (Fig 5C and 5D), the nuclear import of vRNPs [31] (Figs 5E and S7), the viral mRNA synthesis (Fig 5F), and the viral protein expression (Fig 5G) were strongly affected upon regorafenib and sorafenib treatment.

### Regorafenib and sorafenib prevent infection by various viruses

**Fig 6:**
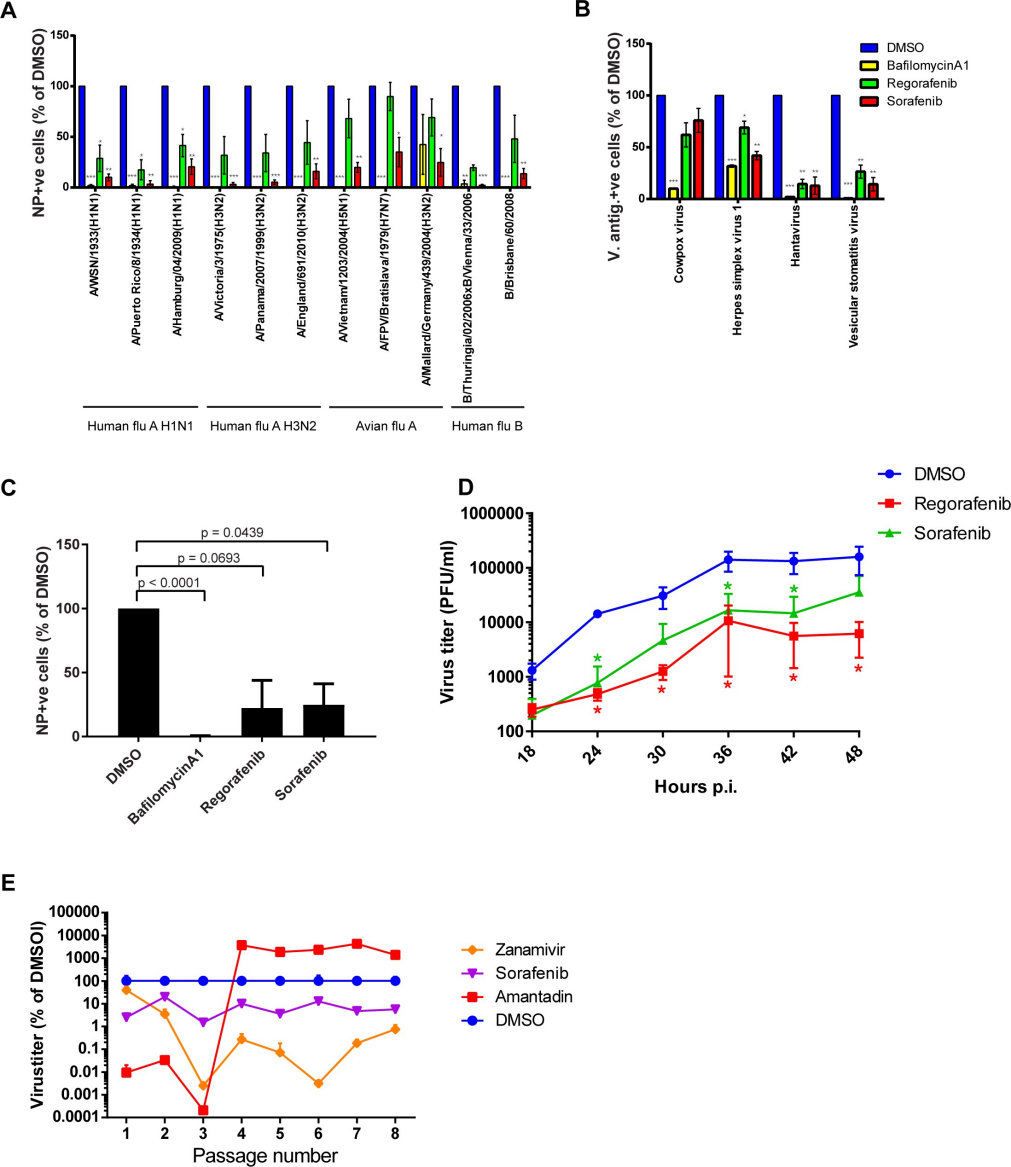
UBKIs possess broad antiviral activity, are effective in primary cells, and impose a barrier against resistance development. (A) A549 cells were pre-treated with 3 μM of small molecules or an equivalent amount of DMSO for 2 h, infected and cultivated for 6 h in presence of small molecules before staining for viral NP protein. Data represent mean ± SEM of n = 3 independent experiments of the fraction of NP-positive cells relative to DMSO. (B) A549 (CPXV, HSV1, VSV) or HEL cell-derived megakaryocyte (HTV) cells were pre-treated as in (A). Cells were infected for 6 h (VSV), 18 h (CHIKV) or 24 h (CPXV, HSV1, HTV) prior to fixation. Presence of viral antigens (HTV: N protein; VSV: NP protein; all other viruses: green fluorescent protein (GFP) was detected by fluorescence microscopy. Data represent mean ± SEM of n = 3 independent experiments of the fraction of viral antigen-positive cells relative to DMSO. Significance in (A) and (B) was calculated based on the one sample t-test for comparison with DMSO control. *: p-value ≤ 0.05, **: p-value ≤ 0.01, ***: p-value ≤ 0.001. (C) Experiments were conducted as in (A) but with hAECB. Zanamivir was applied at 1 μM. (A, B, C) Data represent DMSO-normalized mean ± SEM of n = 3 independent experiments. Indicated p-values based on the one sample t-test describe the difference compared to the DMSO control. (D) hAECB were treated with small molecules at 5 μM. Infected cells were cultivated for 48 h in presence of small molecules and virus load was assessed every six hours by plaque assay. Data represent mean ± SEM of n = 3 technical replicates. Virus titers were analysed by mixed effects models for clustered data where treatment group and time were fixed effects. In the random intercept model residuals associated with different plates were specified to have different residuals. The calculation of p-values revealed a significant decrease of log-transformed virus-titers in the regorafenib and sorafenib-treated groups relative to the DMSO control at the indicated time points: *: p-value ≤ 0.05. (E) MDCK cells were pre-treated with sorafenib (3 μM), zanamivir (0.3 μM), amantadine (3 μM) or an equivalent amount of DMSO for 2 h, infected with MOI 0.001 of IV strain A/England/195/2009–M2_N31S_ and cultivated for 24 h in presence of reagents. Virus titer in tissue culture supernatants was determined and the supernatants were used for inoculation of the next passage. The experiment was terminated after 8 passages. Data represent mean ± SD of technical replicates.

Next, we assessed the breadth of regorafenib’s and sorafenib’s antiviral activity. First, we tested the compounds with a panel of six human and three avian IAVs as well as two IBVs (Fig 6A). Both drugs were active against all strains tested. However, regorafenib only had a moderate, statistically not significant effect on the avian IAVs. We speculated that the differences between the avian and the human IVs were due to differences in the acid stability of the hemagglutinin (HA) proteins: the less stable the HA protein, the higher the pH at which fusion takes place (and the less acidification of the endosomes is required for fusion) [24]. Therefore, we determined the fusion pH for a human IAV (PAN), an IBV (THW), and an avian IAV (A/Mallard/Germany/439/2004(H3N2), MAL) (S8 Fig). To our surprise, the fusion pH for THW and MAL was almost identical and the fusion pH of PAN was even higher. Thus, the HA protein of MAL is not more acid sensitive than that of PAN and THW. The molecular mechanism underlying the low sensitivity of avian IVs against regorafenib thus remains to be illuminated.

Next, we tested the effect of the UBKIs on the non-related viruses cowpox virus (CPXV), herpes simplex virus 1 (HSV1), hantavirus (HTV), and vesicular stomatitis virus (VSV) (Fig 6B). We observed strong effects on HTV, and VSV but only mild effects on CPXV and HSV1. This difference is likely due to the different mechanisms of entry between the viruses: HTV and VSV enter host cells exclusively via the endocytic route [32–34], whereas CPXV and HSV1 can enter by direct fusion with the cellular plasma membrane, as well as via the endocytic route [35, 36]. Of note, we applied both inhibitors at 3 μM, as this dose was neither cytotoxic in A549 cells (Fig 3A and 3B), used with CPXV, HSV1, and VSV, nor in HEL-derived megakaryocytes (S9A Fig), used with HTV. In conclusion, regorafenib and sorafenib display antiviral activity against all viruses tested, particularly those that rely exclusively on the endocytic route.

### Regorafenib and sorafenib inhibit influenza virus replication in murine lung and human primary bronchial epithelial cells and in mice

We next tested if regorafenib and sorafenib are also active in more physiological systems, such as mouse and human primary cells. Both UBKIs showed antiviral activity in mouse lung epithelial cells, however with lowered efficacy and increased cytotoxicity compared to human cells (S10A and S10B Fig). These results indicate that mice may not be an optimal system to investigate the antiviral activity of regorafenib and sorafenib. Still, we assessed the effect of regorafenib on influenza virus replication *in vivo* in BALB/c mice. Regorafenib or vehicle was administered once daily via the oral route. Mice were infected intranasally with 4.4 x 10^4^ PFU/animal, 2 h after the first administration of regorafenib or vehicle, respectively. Two days post-infection, mice were culled, and the viral load in the lungs was determined by plaque assay. In line with the results obtained with mouse lung epithelial cells, we observed a reduced, albeit not significantly changed viral load in the lungs of regorafenib-treated mice (S10C Fig).

Next, we tested the effect of regorafenib and sorafenib on susceptibility of primary human airway epithelial cells from bronchi (hAECB). We used the same dose as with A549 cells, 3 μM, a dose not cytotoxic for hAECB cells (S9B Fig). Also in primary cells, UBKIs efficiently blocked viral protein expression (Fig 6C). Then, we assessed both UBKIs effects on viral replication in hAECB cells. To this end, we employed the highest dose without detectable cytotoxicity to hAECB cells, 5 μM (S9B and S9D Fig). Again, both inhibitors revealed a significant reduction of virus replication, although the effect induced by regorafenib was found to be more pronounced, especially at later time points (Fig 6D).

### Regorafenib and sorafenib impose a high barrier to resistance development in canine cells

Finally, we wanted to test the sensitivity of UBKIs to the development of resistant viruses, and we selected regorafenib and sorafenib as representative UBKIs. To this end, we passaged the IAV strain A/England/195/2009(H1N1) serially in MDCK cells in the presence of 3 μM of sorafenib. In addition, we included amantadine, an inhibitor of the viral M2 protein, and zanamivir, an FDA approved neuraminidase (NA) inhibitor as positive controls within this study. Since nearly all circulating IAV strains are already resistant to the group of adamantanes, we used a recombinant virus mutant that contains a mutation (N31S) within the viral M2 gene that makes the virus susceptible to amantadine treatment [37]. We chose the canine cell line MDCK for the resistance assay as it is a highly efficient IV producer with a very weak IFN response [38], a very important feature for serial passaging experiments. In congruence with previous studies that demonstrated full resistance to DAAs of the group of adamantanes within a few rounds of passaging, we observed resistance against amantadine after 3 passages. However, even after 8 passages no resistance development could be observed when virus replication was inhibited using sorafenib or zanamivir (Fig 6E). To further confirm that resistance to UBKIs is difficult to induce, we also carried out the experiment in MDCK cells infected with A/WSN/1933(H1N1) in the presence of the non-cytotoxic dose (S9C Fig) of 3 μM of regorafenib. Again, after five passages, a time when full resistance to DAAs of the group of adamantanes has normally occurred [39, 40] (and see also Fig 6E), no resistance to regorafenib was observed (S11 Fig).

## Discussion

We present here a large-scale comparative loss-of-function screen for IV replication. We also included an avian IAV strain and an IBV strain. An avian strain has only been tested in one other published screen [13], whereas no IBV strain was in the focus of previous loss-of-function screens. Due to the comparative nature of the present screen, we were able to detect strain-specific differences in host factor requirement. The number identified, several hundred strain-specific genes and gene groups, was unexpectedly large. The impact of these genes and gene groups on viral replication differed in many instances either between the three human IVs versus the avian IV strain and between the three IAVs versus the IBV strains. These differences likely reflect evolutionary divergence (IAV vs. IBV) or adaptation to divergent hosts (human IV vs. avian IV).

Apart from identifying differences between IVs, the present study was designed to identify genes and cellular processes which are conserved parts of the viral replication cycle across the different types of IVs. We identified 133 host cell genes that are strictly required for replication of all four IV strains tested. Among these strain-independent genes, those involved in nucleocytoplasmic transport, cell cycle regulation, RNA splicing, translation, and tRNA charging were highly enriched.

While several of these gene requirements have been previously noted [12], tRNA charging has not been seen in previous screens of human IAVs [12]. The exact implication of tRNA charging in IV replication remains to be characterized. Yet, it might affect translation since charged tRNAs constitute the substrates for protein synthesis. According to our results, the cellular process most strictly required for IV replication across all strains is the nucleocytoplasmic transport. Therefore, agents blocking nucleocytoplasmic transport represent attractive future candidates for a host-directed therapy of influenza. In accordance with this, promising results have been published with the exportin 1 inhibitor verdinexor [41]. However, a recent study demonstrated that IAVs quickly become resistant to depletion of importin-α7, another factor involved in nucleocytoplasmic trafficking, when an in vivo model is used and that the resistant virus even gained higher virulence [42]. Therefore, in vitro and in vivo experiments are required to further explore the therapeutic potential of cellular factors in general as antiviral drug target.

The *de novo* development of drugs is a long and expensive process, making the repurposing of pharmaceuticals for new indications an attractive strategy. By drug database screening, we identified several approved or experimental drugs targeting strain-independent host factors. Most of these drugs were screened in A549 cells for antiviral activity and cytotoxicity rendering fourteen with a potential for therapeutic application. We focused on the FLT4 inhibitors regorafenib and sorafenib and found that in addition to VEGFR3 at least three other RTKs (FGFR1, VEGFR1, PDGFRB) targeted by these drugs are relevant to IV replication. Therefore, the antiviral activity of regorafenib and sorafenib is likely to be due to the inhibition of several target proteins in parallel, and may even include further kinases. Both drugs interfered with fusion of the viral and the endosomal membranes. Impairment of an early step in the IV life cycle, such as fusion, necessarily would affect all downstream steps. Consequently, it remains an open question if regorafenib and sorafenib affect additional (post-fusion) steps of viral replication. Interestingly, the broad-range RTK inhibitor genistein as well as a broad-range RTK inhibitor mix (containing the VEGFR/PDGFR inhibitor SU4312), have been shown to inhibit the uptake of IV virions [43], indicating that while some RTKs are required for internalization, others are required for fusion. It is possible that regorafenib and sorafenib inhibit kinases involved in endosomal maturation or block kinases that phosphorylate the V-ATPase subunits, since their phosphorylation status regulates their activity and thus endosomal acidification [44, 45]. Future studies will shed more light on the mechanisms involved in the UBKI-mediated block of fusion.

Regorafenib and sorafenib not only affected IV but also HTV, VSV, and HSV1. The effects on viruses using the endocytic route were stronger than the effect on those mainly fusing directly with the plasma membrane. The effect of regorafenib on avian IV was milder than on human IV. This was unexpected, since we incorporated regorafenib in our small molecule screen because one of its targets, FLT4, was universally required in the siRNA screen. Possibly, other targets of regorafenib (s. above) are strain-specific. The molecular mechanism underlying the differential effect of regorafenib on human and avian IVs is not clear but appears to be independent of the fusion pH of the HA protein of the different viruses. Recent studies showed that prior to fusion, pH-dependent conformational rearrangements–especially of the M1 protein–take place within the lumen of the virion, and these rearrangements are important for efficient uncoating [46, 47]. As these processes require luminal acidification, they are dependent on the activity of the viral M2 ion channel. Thus, the IV strain-specific effect of regorafenib might be due to differences in the M1 or M2 proteins. Another possibility is that, as discussed above, regorafenib impairs steps in the viral replication cycle downstream of fusion, and in a strain-specific manner. Nevertheless, due to the broad antiviral activity, regorafenib and sorafenib might be suited for a host-directed therapy of various viral diseases. This indicates a clear benefit compared to current influenza treatments, which are only effective against IV. In addition, both drugs efficiently inhibited IV replication in primary human respiratory cells, implying that our findings might indeed have a physiological relevance.

A recent study investigated the impact of 273 kinase inhibitors on IAV replication and reported only intermediate effects for regorafenib and sorafenib [48]. The cause of this discrepancy is not clear but might be due to differences in the assay setup. Furthermore, the previous study used an avian IV strain, and avian IVs are less sensitive to regorafenib treatment according to our results. In line with our findings, sorafenib mono-treatment has previously been found to impair replication of various viruses such as adenovirus, coxsackievirus, enterovirus 71, hepatitis C virus, human cytomegalovirus, mumps virus, polyomavirus BK, Rift Valley fever virus, and West Nile virus, respectively, and combinatorial treatment with sildenafil led also to inhibition of dengue virus, rabies virus, and yellow fever virus [49–55]. Furthermore, regorafenib treatment has been reported to affect adenovirus, coxsackievirus, and mumps virus, as well [54]. Interestingly, different mechanisms of antiviral activity have been described, including inhibition of entry, viral protein translation, and viral egress [50, 51, 55]. An effect of these drugs on viral fusion has, to our knowledge, not been reported so far. In conclusion, our study and the existing literature support the notion that regorafenib and sorafenib possess (A) broad antiviral activity and (B) different mechanisms of antiviral activity.

Surprisingly, the efficacy of regorafenib and sorafenib in primary cells from bronchi was much lower than in the pneumocyte-derived cell line A549. This difference might be related to the different origin of the cell types (bronchi versus alveoli) but also to the different degrees of nativeness (primary cells versus cell line). To explore this further, the effect of these drugs on IV replication should be tested in additional relevant primary cell types (from upper respiratory tract and alveoli), followed by a suitable *in vivo* model of IV replication. In an initial animal experiment, regorafenib showed a rather weak antiviral effect in mice. However, regorafenib also exhibits a considerably lower efficacy *in vitro* in murine compared to human cells. Thus, the preliminary *in vivo* data might underestimate regorafenib’s true *in vivo* efficacy in a system which resembles the human host more closely, e.g. the ferret [56]. Consequently, experiments with such highly authentic animal models need to be performed in a future study to better judge the clinical applicability of regorafenib and sorafenib.

Importantly, we did not observe development of resistance against regorafenib and sorafenib even after up to eight passages in cell culture. This indicates that UBKIs impose a strong barrier towards the development of viral resistance, exceeding the threshold of the viral M2 ion channel-directed drug amantadine, which occurs after three to five passages shown by us and others [39, 40, 57]. Interestingly, the passaged virus, that gained resistance to amantadine, now replicates at a higher rate compared to the initial virus. The mutation probably increases viral fitness slightly, in line with the fact that all currently circulating pH1N1 viruses are resistant to amantadine, despite a lack of selection pressure since amantadine is no longer used for therapy [58]. In contrast, even after eight passages development of resistance to approved viral NA inhibitors, such as zanamivir, was neither observed by us nor are has it been reported by others, to our knowledge. To compare the resistance profiles of viral NA inhibitors and regorafenib/sorafenib, long-term studies need to be performed.

The current gold standard in influenza therapy are the DAAs oseltamivir and zanamivir, which target the viral neuraminidase. The IC_50_ of these drugs is in the low nanomolar range [59]. We observed an IC_50_ of 3 nM for zanamivir in our experimental system, which outperforms regorafenib and sorafenib. However, oseltamivir and zanamivir lack activity against unrelated viruses. Furthermore, resistance against oseltamivir and zanamivir has been reported in the clinic [3–5]. Thus, regorafenib and sorafenib might represent a valuable alternative to the current standard.

An existing uncertainty of sorafenib as host-directed therapeutic against influenza is its immune modulatory effects. An impairment of dendritic cell differentiation and function has been observed [60, 61]. In case of T cells and natural killer cells, inhibitory but also stimulatory effects have been reported [62–65]. Nevertheless, the seroprotection rates induced by influenza vaccination do not differ between cancer patients undergoing sorafenib treatment and controls [66]. The effects of regorafenib on the immune response have not been thoroughly characterized yet. Although interference with the immune response represents a potential disadvantage for an antiviral drug, its use might nonetheless be beneficial as long as the antiviral effects significantly overcompensate for the immunomodulatory effects. This, however, can only be addressed in suitable animal models.

For approval of cancer therapeutics, such as regorafenib and sorafenib, more severe adverse events are tolerable than for influenza drugs considering influenza is usually not lethal. Although the therapeutic margin of regorafenib/sorafenib is indeed lower compared to standard anti-influenza drugs, such as zanamivir, it is still larger than that of ribavirin, a drug approved for treatment of several virus infections [67]. The most prominent side effects of regorafenib and sorafenib are rash and hypertension. However, both UBKIs are usually administered over weeks or months during cancer therapy, whereas a few days might be sufficient in case of influenza treatment. Although short-term treatment would not necessarily prevent any occurrence of adverse events, the duration of side effects would be shortened and the impairment of patients’ quality of lives lowered compared to cancer treatment. In addition, it might be possible to apply both UBKIs locally by inhalation, in contrast to the systemic administration used for cancer therapy, which would allow lower dosing (relative to the bodyweight) and thus potentially reduce adverse events. Furthermore, especially in cases of severe influenza or acute respiratory distress syndrome, the potential side effects of regorafenib/sorafenib may be acceptable. With the standard dosing regimen (160 mg, once daily), the plasma concentration of regorafenib reaches a maximum of 3.6 μM and stays above the IC_50_ for IAV replication in A549 cells (i.e. > 0.89 μM) for at least 48 h [68]. Standard administration of sorafenib (400 mg, twice daily) achieves a plasma concentration of approximately 4 μM, which is stable for at least 24 h [69]. Thus, there is reason for confidence that regorafenib and sorafenib are viable treatment options.

In summary, our siRNA screening campaign identified nucleocytoplasmic transport as the host cellular process that is most strictly required for replication across all tested IV strains. Subsequent small molecule screening showed that the two UBKIs regorafenib and sorafenib have powerful broad range antiviral activity, are also efficacious in primary cells, and impose a barrier towards the development of resistant virus strains.

## Materials & methods

### Ethical permissions

All animal work was conducted in accordance with European regulations and approved by the Berlin state authorities, Landesamt für Gesundheit und Soziales (Reg No: G0206/12 and 0321/08).

### Cells

A549 adenocarcinomic human alveolar basal epithelials (ATCC CCL-185) were normally grown in DMEM media (Thermo Fischer) supplemented with 2 mM L-glutamine, 1 mM sodium pyruvate, 100 U/ml penicillin/streptomycin (P/S) and 10% heat-inactivated fetal calf serum (hiFCS, Biochrom;) (DMEM complete medium). A549 cells to be infected with CPXV or HSV1 were grown in absence of sodium pyruvate. A549-IFN-Luc cells were generated by lentiviral transduction using the Cignal Lenti ISRE Reporter (luc) Kit CLS-008L (Qiagen) and grown in DMEM complete medium supplemented with 5 μg/ml puromycin dihydrochloride (Thermo Fischer). A549-CRISPR/Cas9 knockout cells were generated as described below and grown in DMEM complete medium supplemented with 10 μg/ml blasticidine and 2.5 μg/ml puromycin. The primary human airway epithelial cells from bronchi (hAECB, Epithelix Sarl) were propagated using NIH/3T3 cells gamma-irradiated with 30 Gy as feeders, using a 3:1 mixture of Ham’s F-12 nutrient mix (Invitrogen) and DMEM supplemented with 5% hiFCS, 0.4 μg/mL hydrocortisone (Sigma-Aldrich), 5 μg/mL recombinant human insulin (Sigma-Aldrich), 8.4 ng/mL cholera toxin (Sigma-Aldrich), 24 μg/mL adenine (Sigma-Aldrich), 10 ng/mL recombinant human EGF (Thermo Fischer) and 9 μM Y27632 (Miltenyi Biotec). When hAECB reached 80 to 90% confluence, they were separated from feeders by differential trypsinization and passaged to new culture vessels with CnT-Prime Airway medium (CELLnTEC). HEL 92.1.7 human erythroleukemia cells (HEL cells, ATCC TIB-180) were grown in RPMI 1640 media (Thermo Fischer) supplemented with 2 mM L-glutamine, 25 mM HEPES, 100 U/ml P/S and 10% hiFCS and differentiated into megakaryocytes at infection using stimulation with phorbol myristate acetate [70]. MDCK Madin-Darby canine kidney epithelial cells (ATCC CCL-34) and Vero E6 African green monkey kidney epithelial cells (ATCC CRL-1586) were grown in DMEM complete medium without sodium pyruvate. MLE 12 SV40-transformed mouse lung epithelial cells (ATCC CRL-2110) were grown in DMEM/Ham’s F10 media (Thermo Fischer) supplemented with 2 mM L-glutamine, 50 nM hydrocortisone, 5 μg/ml insulin, and 2% hiFCS. NIH/3T3 mouse fibroblast cells (ATCC CRL-1658) were grown in DMEM complete medium lacking antibiotics. All cells were grown at 37°C under humidity and 5% CO_2_ on cell culture-treated plastic ware unless stated otherwise.

### Generation of A549-CRISPR/Cas9 knockout cell lines

First, A549 were transduced with lentiviruses based on the plasmid lentiCas9-Blast (Addgene number: 52962). After selection with blasticidine for 10 days, cells were transduced with lentiviruses derived from the plasmid lentiGuide-Puro [71] (Addgene number: 52963) that leads to the expression of a guideRNA with gene dependent targeting sequences (S12 Table). Finally, single cell clones were kept under selection medium containing 10 μg/ml blasticidine and 2.5 μg/ml puromycin for an additional 10 days.

### Viruses

Influenza virus strains A/Puerto Rico/8/1934(H1N1), A/Panama/2007/1999(H3N2), A/Mallard/Germany/439/2004(H3N2) (all provided by T. Wolff, Robert Koch Institute, Germany), A/Hamburg/04/2009(H1N1) (provided by S. Becker, University of Marburg, Germany), A/Victoria/3/1975(H3N2) (described recently in [72]), A/FPV/Bratislava/1979(H7N7) (provided by S. Ludwig, University of Münster, Germany), B/Thuringia/02/2006xB/Vienna/33/2006 (provided by Avir Greenhills Biotechnology, Austria, [73]), and B/Brisbane/60/2008 (provided by T. Frensing, Max Planck Institute for Dynamics of Complex Technical Systems, Germany) were grown in the allantoic cavities of 9- or 10-day-old embryonated chicken eggs. Seeds of influenza virus strains A/WSN/1933(H1N1) (provided by St. Jude Children’s Research Hospital, USA) and A/Vietnam/1203/2004(H5N1) (provided by Y. Kawaoka, University of Wisconsin-Madison, USA) were produced by reverse genetics as described in [74] and [75], respectively. Virus seeds were subsequently propagated in the allantoic cavities of 9- or 10-day-old embryonated chicken eggs and used for experimentation. The amantadine sensitive A/England/195/2009–M2_N31S_(H1N1) virus was generated using reverse genetics. A plasmid-based system that allows rescue of a prototypic first wave pH1N1 virus A/England/195/2009 [76] was employed. The plasmid encoding segment 7 vRNA was mutated to generate an amino acid change in the M2 coding region at amino acid 31 from N to S. Influenza virus strain A/England/691/2010(H3N2) was obtained through the MOSAIC consortium [76]. A/England/195/2009(H1N1) were described recently. Vesicular stomatitis virus strain Indiana (provided T. Wolff) was grown in MDCK cells. Recombinant, GFP expressing viruses cowpox virus strain Brighton Red (provided by K. Tischer, Freie Universität Berlin, Germany), herpes simplex virus 1 strain KOS K26GFP (provided by P. Desai, John Hopkins University, USA), and hantavirus strain Hantaan 76–118 were grown in Vero E6 cells. For experiments, virus stocks were diluted to the desired MOI in PBS containing 9 μM bovine serum albumin (BSA), 492 μM CaCl_2_, and 901 μM MgCl_2_ (infection buffer), unless stated otherwise.

### siRNA screen execution

A549 wildtype or A549-IFN-Luc cells were transfected with siRNAs in 384-well format as previously described for A549 wildtype cells [11]. After 24 h, the A549-IFN-Luc cells were subjected to IFN induction assay as described below. After 48 h, the A549 wildtype cells were either subjected to cell viability assays (WST-1 assay, cell number determination assay) or virus replication assay as described below. All multi-well pipetting steps were performed using a Biomek FX^P^ Laboratory Automation Workstation (Beckman Coulter). The siRNA library was composed of a validated human kinome library and a custom designed siRNA library (Qiagen) in total containing 3,482 siRNAs targeting 1,208 human genes. On each plate, control RNAs were included: non-targeting siRNA Allstars (14 wells) as negative control for all assays, polyinosinic-polycytidylic acid (poly I:C, final concentration: 2 μg/ml, 6 wells, poly(I:C)-LMW, Invivogen) as positive control for the IFN induction assay, an siRNA targeting the cellular gene PLK1 (siPLK1, 4 wells, [11]) as positive control for cell viability assays, and an siRNA targeting the IAV NP gene in case of IAV (8 wells, [77]) or an siRNA targeting the cellular gene XPO1 (8 wells, target sequence: 5’-CTGTGTCAGTTTGTAATGGAA-3’) in case of IBV, respectively, as positive control for virus replication assays. All siRNAs were purchased from Qiagen.

### IFN induction assay

Twenty-four hours post-transfection, tissue culture supernatants of the A549-IFN-Luc cells, an A549 derivative cell line stably expressing *photinus pyralis* luciferase under control of tandem repeats of the IFN stimulated response element, were replaced with Beetle Lysis-Juice luciferase substrate (p.j.k.) and luciferase activity was quantified using the EnVision Multilabel Reader (Perkin Elmer).

### WST-1 assay

In siRNA-transfected A549 cells, WST-1 assay was performed 48 h post transfection. To assess cytotoxicity of small molecules, cells were washed with PBS and incubated for 36 h (A549) or 48 h (HEL-derived megakaryocytes, hAECB, MDCK, MLE 12,). Cells were washed with infection buffer and cultured for 30 min in presence of DMEM supplemented with 9 μM BSA, 2 mM L-glutamine, 1 mM sodium pyruvate, 100 U/ml P/S, 25 mM HEPES (cell culture grade) (infection medium) containing 10% of WST-1 reagent (Roche). Finally, light absorbance at 460 nm and 590 nm was quantified using the EnVision Multilabel Reader and cell viability was defined as the difference of absorbance at 460 nm and 590 nm.

### Cell number determination assay

To determine the number of cells per well, cells subjected to WST-1 assay as described above were immediately fixed and nuclei stained by incubation with 3.7% formaldehyde containing 10 μg/ml of Hoechst for 1 h. The solution was replaced with PBS and the number of nuclei per well was quantified using the Acumen eX3 Cytometer (TTP Labtech).

### Virus replication assay (fluorescent focus assay)

To test the effect of siRNAs, virus replication assay was started 48 h post-transfection. To test the effect of small molecules, cells were washed with PBS and incubated for 2 h with small molecules under normal culture conditions. To test the effect of CRISPR/Cas9-mediated gene deletion, A549-CRISPR/Cas9 cells were subjected to the assay 10 days post selection. Then, cells were inoculated with virus diluted in infection buffer. Transfected A549 cells were inoculated with an MOI of 0.01 (WSN, PAN), 0.04 (VN), or 2.6 (THW). Small molecule-treated A549 and MLE 12 cells were inoculated with WSN with an MOI of 0.02, A549-CRISPR/Cas9 cells were inoculated with WSN with an MOI of 0.005 and MDCK cells were inoculated with WSN with an MOI of 0.001. After 40 min, plain infection medium or infection medium containing small molecules, respectively, containing 1 μg/ml TPCK-treated trypsin (Sigma-Aldrich) were added, and the cells were incubated under normal culture conditions for 24 h (WSN in MDCK), 36 h (WSN in all A549-derived cells, PAN, VN), 48 h (WSN in MLE 12), or 96 h (THW). Virus load was determined by inoculating (new) MDCK cells with the supernatants followed by fluorescence microscopic analysis of infection. To this end, MDCK cells were washed and inoculated with undiluted supernatant for 1 h. Virus was removed and MDCK cells were cultivated for 6 h under normal culture conditions with infection medium. Cells were stained for viral NP protein and nuclei and the infection rate (i.e. the rate of NP positive cells) determined as described previously [11]. The final read-out of the assay was the virus titer. This was extrapolated from the infection rate based on the following formula: multiplicity of infection = -ln(1- infection rate).

### siRNA screen data analysis

Raw data from the individual assays were analyzed with R (http://www.R-project.org/) using the cellHTS2 package using either “normalized percent inhibition” (NPI; used for analysis of IFN induction and cell viability screens and virus replication screens to identify strain-independent genes and processes) or “percent of control” (POC; used for analysis virus replication screens to identify strain-specific genes and processes) for raw data normalization [78]. For NPI normalization, raw data were scaled relative to an inactive and an active siRNA as described in ‘siRNA screen execution’. For POC normalization, raw data were scaled only relative to non-targeting siRNA Allstars. Screen results were analyzed based on three replicates. To ensure high data quality, only replicate plates with a Pearson product-moment correlation coefficient ≥ 0.45 and a background-to-signal ratio’ of ≥ 5 were analyzed. The background-to-signal ratio’ was calculated by dividing the plate’s average raw data value for the inactive control siRNA (Allstars) by the value of the respective active control (i.e., poly I:C, or anti-PLK1, -NP, or -XPO1 siRNA). IFN induction and cell viability assays were only analyzed on siRNA level. For analysis of virus replication screens, siRNA results were summarized on gene level using the collective mean and cSSMD estimated by the method of moments [18].

As we did not observe a correlation of signals in the IFN induction assay and the virus replication assay (using the siRNA level data of the WSN screen, S12 Fig), we regarded all sample siRNAs as non-inducers of IFN. The cut-off for cytotoxic siRNAs according to WST-1 assay was selected based on the point closest to (1,1) of a receiver operating characteristic curve using the siRNA level data of the WST-1 and the virus replication assay with IV strain WSN for Allstars and siPLK1. The cut-off for cytotoxic siRNAs according to the cell number determination assay was set as the mean—3 x SD of the cell count for Allstars.

In contrast to the IFN induction, WST-1, and cell number determination assays, virus replication screens were not analyzed on siRNA but on gene level. For identification of genes required by all four IVs, NPI-normalized input data were used. Genes with collective mean > 0.5 and cSSMD ≥ 1 were regarded as proviral. Genes with collective mean < -1 and cSSMD ≤ 1 were regarded as antiviral. All other genes were regarded as neutral. Genes identified as proviral with all four IV strains tested are designated “strain-independent genes”.

### Gene group enrichment analyses

All strain-independent genes were subjected to gene group enrichment analysis using IPA (Ingenuity H Systems) and DAVID [19]. In DAVID, databases GOTERM_BP_ALL, GOTERM_CC_ALL, GOTERM_MF_ALL, BIOCARTA, KEGG_PATHWAY, and REACTOME_PATHWAY were collectively analyzed by Functional Annotation Clustering (highest stringency).

### Identification of strain-specific genes and pathways

POC-normalized data were analyzed for host cell genes and pathways with virus strain-specific relevance with a mixed effects model using R with the library _nlme (http://CRAN.R-project.org/paclage=nlme>). Genes were assigned gene groups according to databases Gene Ontology database [20], the Kyoto Encyclopedia of Genes and Genomes database [21], and the Reactome database [22], and only groups represented by ≥ 2 genes were analyzed. Genes/gene groups with a false discovery rate-adjusted p-value ≤ 0.05 for the interaction between the factors “virus” and “treatment” were regarded to be differentially required between the tested viruses. Differentially required entities with a POC-score ≥ 2 for all four and differentially required entities not classified as pro- or antiviral for any of the four viruses, respectively, were excluded from further analyses and the remaining genes and gene groups were designated “strain-specific genes” and “strain-specific pathways”, respectively. Strain-specific genes were furthermore analyzed with the clustering algorithm CLICK [79] embedded in EXPANDER [80].

### In silico screen for candidate drugs via a drug repositioning approach

All information about drugs targeting any of the 133 strain-independent genes was retrieved with Ingenuity Pathway Analysis. No drug combinations were investigated.

### Small molecules

Small molecules tested as IV inhibitors were dissolved in DMSO if not stated otherwise. Abiraterone acetate, ketoconazole, vemurafenib, axitinib, cabozantinib, pazopanib, pazopanib HCl, vandetanib, ruxolitinib, tofacitinib, tacrolimus, temsirolimus, clopidogrel, ticagrelor, and ticlopidine were gifts from J. von Kries (Leibniz-Institut für Molekulare Pharmakologie, Germany). Alvocidib, selumetinib, trametinib, talmapimod, everolimus, OSI-027, pimecrolimus, sirolimus, epoprostenol, pictrelisib, SF 1126, and XL147 were purchased from Santa Cruz Biotechnology. Abiraterone, motesanib, regorafenib, tivozanib, and buparsilip were purchased from TRC. Dasatinib, prasugrel, treprostinil, and idealisib were purchased from Cayman Chemical. Sorafenib, sunitinib, and PX-866 were purchased from US Biological. Amantadine hydrochloride and zanamivir (Relenza), both dissolved in water, were purchased from Sigma-Aldrich and GlaxoSmithKline, respectively. Bafilomycin A1 was purchased from Adipogen. Ridaforolimus was purchased from BioAustralis. Dactolisib was purchased from Biovision. Zanamivir was purchased from GlaxoSmithKline and dissolved in water. Dynasore (dissolved in DMSO) and importazole (dissolved in water) were purchased from Sigma-Aldrich. For experimental analysis, small molecules were diluted to final concentration in infection medium (WST-1 assay, mRNA expression analysis, analysis of primary infection (IVs, VSV), virus replication assay), infection medium supplemented with 0.1% hiFCS (analysis of attachment, internalization, fusion, uncoating, and vRNP import), DMEM complete medium (analysis of primary infection of CPXV and HSV1), RPMI 1640 medium supplemented with 2 mM L-glutamine, 25 mM HEPES, 100 U/ml P/S and 10% hiFCS (analysis of primary infection of HTV), or DMEM complete medium containing only 0.5% hiFCS (starvation medium) (analysis of intracellular EGF and transferrin levels).

### Determination of antiviral potency and cytotoxicity of small molecules

The antiviral potency and cytotoxicity of small molecules in A549 cells were tested in 384-well format, and all multiwell pipetting steps were performed using a Biomek FX^P^ Laboratory Automation Workstation. The antiviral potency and cytotoxicity of small molecules in MLE 12 cells were tested manually in 96-well format. Small molecules were three-fold serially diluted in infection medium generating eight different concentrations. Then, small molecules were applied to the cells and the assays were performed as described above. EC_50_ values for A549 cells were calculated using the R software package drc [81]. EC_50_ values for MLE 12 cells were calculated using Prism (GraphPad Software; http://www.graphpad.com/). To determine cytotoxicity of small molecules in hAECB, cells were treated as described for MLE 12 cells.

### Quantification of viral load in cell culture supernatants by plaque assay

To test the effect of small molecules on the viral load of cell culture supernatants, cells were washed with PBS and incubated for 2 h with small molecules under normal culture conditions. Then, cells were inoculated with an MOI of 0.001 of WSN diluted in infection buffer. After 40 min, infection medium containing small molecules containing 1 μg/ml TPCK-treated trypsin (Sigma-Aldrich) were added, and the cells were incubated under normal culture conditions for up to 48 h. In case of therapeutic small molecule application, cells were not pre-treated with small molecules prior to infection, but small molecules were added 4 h post infection. Virus load was determined by plaque assay on MDCK cells using an agar overlay medium [82].

### Analysis of primary infection

To analyze primary infection, A549, and HEL cells as well as hAECB were washed with PBS and incubated for 2 h with small molecules (3 μM) or an equivalent of DMSO under normal culture conditions. Then, cells were inoculated with an MOI of 0.1 (CPXV, HSV1, VSV), 0.3 (IVs), or 1.5 (HTV), respectively, for 30 min at 4°C. Unbound virus was removed and the cells cultivated for 6 h (IV, VSV) or 24 h (CPXV, HSV1, HTV) in presence of small molecules. Then, the rate of infected cells was determined by microscopically quantifying the number of cells positive for viral antigen. In case of IVs and VSV, presence of the NP protein was investigated by indirect immunofluorescence analysis using primary antibodies MCA400 (IAVs, AbD Serotec), MCA403 (IBVs, AbD Serotec), polyclonal-anti hantavirus N protein and V5507 (VSV, Sigma-Aldrich). In case of CPXV and HSV1, presence of GFP was investigated by fluorescence analysis. Microscopic analysis was performed as described previously [11].

### Labeling of IV with Alexa Fluor 647

500 μg/ml concentrate of IV strain PAN were mixed with PBS up to 50 μl and 2 ng AlexaFluor647-NHS-Ester (Thermo Fischer) and incubated over night at 4°C on a shaker at 300 rpm. To separate virus and dye, virus was precipitated by centrifugation (25,000 x g, 15 min, 4°C) and the pellet was resuspended in 100 μl PBS. Finally, virus was filtered using a 0.45 μm Filter Unit (EMD Millipore).

### Analysis of virus attachment

A549 cells were washed with PBS and pre-incubated for 30 min with small molecules (3 μM), an equivalent of DMSO, or with 1.5 U/ml of neuraminidase from *Clostridium perfringens* (Sigma-Aldrich), respectively, under normal culture conditions. Cells were inoculated for 1 h at 4°C with 0.002 hemagglutination units per cell of IAV strain PAN labeled with Alexa Fluor 647 dye as described in ‘Labeling of IV with Alexa Fluor 647’. After removing unbound virus by washing with ice-cold PBS twice, cells were analyzed for virus attachment either qualitatively by fluorescence microscopy or quantitatively by flow cytometry. For quantitative analysis, cells were harvested by scraping and precipitated by centrifugation (1200 x g, 5 min, 4°C). For both analyses, cells were fixed by incubation with 4% paraformaldehyde. For image acquisition, nuclei were stained with DAPI and mounted in Mowiol (Sigma-Aldrich), whereas samples for flow cytometry analyses were precipitated and resuspended in PBS again. Finally, either representative images were acquired on an inverted FV-1000 MPE laser scanning microscope (Olympus) using a 60x UPlanSApo water objective (numerical aperture 1.2) (Olympus) or Alexa Fluor 647 intensity was determined using a FACSAria II flow cytometer (Becton Dickinson). Three independent experiments were performed in duplicates and for each duplicate measurement 10,000 cells were analyzed by flow cytometry.

### Analysis of virus internalization

A549 cells were pre-treated with small molecules and infected as described in ‘Analysis of virus attachment’. Dynasore was applied at 100 μM. One hour after inoculation, cells were incubated in presence of small molecules for an additional 45 min under normal culture conditions. Then, cells were analyzed for virus internalization either qualitatively by fluorescence microscopy or quantitatively by flow cytometry. For quantitative analysis, cells were harvested by scraping, precipitated by centrifugation (1200 x g, 5 min, 4°C) and fixed with 4% paraformaldehyde. For both analyses, cells were blocked with PBS containing 3% BSA for 2 h at room temperature. Subsequently, the cells were incubated with an anti-H3N2 HA protein antiserum (ViroStat) diluted 1:500 in PBS containing 1% BSA at 4°C overnight, followed by incubation with an Alexa Fluor 488-labeled secondary antibody (Thermo Fischer) diluted 1:1000 in PBS containing 1% BSA at room temperature for 3 h. Finally, either representative images were acquired as described for ‘Analysis of virus attachment’ (qualitative analysis) or Alexa Fluor 647 and Alexa Fluor 488 intensities were determined using a FACSAria II flow cytometer (Becton Dickinson) (quantitative analysis). Three independent experiments were performed in duplicates and per duplicate measurement, 10,000 cells were analyzed by flow cytometry.

### Analysis of intracellular epidermal growth factor (EGF) and transferrin levels

A549 cells were serum-starved for 3 h by incubation with starvation medium. Then, cells were incubated for 30 min with small molecules (dynasore: 100 μM, regorafenib/sorafenib: 3 μM) or an equivalent of DMSO, respectively, under normal culture conditions. Afterwards, cells were transferred to 4°C and washed twice with cold PBS and incubated with 100 ng/ml Alexa Fluor 647-labeled EGF (Thermo Fischer) and 25 μg/ml Alexa Fluor 488-labeled transferrin (Thermo Fischer) diluted in starvation medium for 1 h at 4°C. Cells were pulsed for 10 min at 37°C, washed three times with PBS and chased for different periods of time (0, 30, 60, and 120 min) in starvation medium containing small molecules but lacking EGF and transferrin. After the chase period, cells were transferred to 4°C and washed once with cold acid buffer (150 mM NaCl, 25 mM NaOH, pH 3.5) to remove surface-bound EGF and transferrin. Then, cells were washed twice with cold PBS, harvested by scraping and precipitated by centrifugation (1200 x g, 5 min, 4°C). Finally, cells were fixed with 4% paraformaldehyde and analyzed for Alexa Fluor 647 and Alexa Fluor 488 intensities as described for ‘Analysis of virus attachment’. Three independent experiments were performed in duplicates and per duplicate measurement, 10,000 cells were analyzed by flow cytometry.

### Analysis of fusion

A549 cells grown in glass-bottom dishes (35 mm, uncoated, MatTek Cooperation) were washed once with PBS and pre-incubated with small molecules for 30 min (bafilomycin A1: 200 nM; regorafenib and sorafenib: 3 μM) or an equivalent of DMSO, respectively, and Hoechst under normal culture conditions. Then, cells were inoculated at 4°C with 62.5 μg/ml of IAV strain PAN simultaneously labeled with DiOC18 and DiI as described previously [29]. After 15 min, the cells were washed three times with cold PBS and subsequently cultivated under normal culture conditions in the presence of small molecules. Cells were live-imaged for 65 min as described for ‘Analysis of virus attachment’. For quantification, images of three independent experiments at indicated time points were summarized on the z-axis and particle recognition was performed using ImageJ (http://imagej.nih.gov/). Per image, 60 to 80 cells, containing 300 to 500 particles in total, were analyzed for changes in DiOC18 intensity over time.

### Analysis of uncoating and vRNP import

A549 cells grown on glass slides were washed with PBS and pre-incubated for 30 min with small molecules (3 μM) or an equivalent of DMSO, under normal culture conditions. Then, cells were transferred to 4°C, washed twice with cold PBS, and inoculated with an MOI of 5 of IAV strain PAN. After 1 h, cells were washed three times with cold PBS and incubated for 3 h (uncoating) or 4.5 h (vRNP import) in the presence of small molecule inhibitors and 100 μM cycloheximide (Sigma-Aldrich) under normal culture conditions. Then, cells were fixed by incubation with 4% paraformaldehyde for 20 min at room temperature, permeabilized with PBS containing 0.5% Triton X-100 for 15 min, and blocked with PBS containing 3% BSA for 4 h. Cells were incubated overnight at 4°C with primary antibodies targeting either the IAV M1 (MCA401, AbD Serotec) or NP (EMD Millipore) proteins, respectively, diluted 1:500 in PBS containing 1% BSA, followed by a 5 h incubation step with an Alexa Fluor 488-labeled secondary antibody at room temperature. Finally, nuclei were stained by 30 min incubation with DAPI at room temperature and the slides mounted in Mowiol (Sigma-Aldrich). Fluorescence microscopic analysis was conducted as described for ‘Analysis of virus attachment’. Images of uncoating assay were segmented with CellProfiler 2.1.1 (http://cellprofiler.org/) and classification of the subcellular distribution of the M1 protein (i.e. dispersed vs. dot-like) was performed using the CellProfiler Analyst 2.0 (http://cellprofiler.org/). Three independent experiments, performed in duplicates, were subjected to analysis, and per experiment approx. 400 cells were investigated.

### mRNA expression analysis

A549 cells were washed with PBS and pre-incubated for 2 h with small molecules (3 μM) or an equivalent of DMSO under normal culture conditions. Then, cells were inoculated with an MOI of 0.3 of IAV strain WSN for 30 min at 4°C. Subsequently, unbound virus was removed and the cells were cultivated for 4 h in the presence of small molecules. Total RNA was isolated from the cells using the GeneJET RNA purification kit (Thermo Fischer) according to the manufacturer’s instructions. For reverse transcription, oligo(dT) primer was used. Resulting cDNAs were quantified by quantitative PCR with oligonucleotides specific for viral NP mRNA (5’-CTCGTCGCTTATGACAAAGAAG-3’ and 5’-AGATCATCATGTGAGTCAGAC-3’), viral M1 mRNA (5’-GACCAATCCTGTCACCTC-3’ and 5’-GATCTCCGTTCCCATTAAGAG-3’), viral M2 mRNA (5’-GAGGTCGAAACGCCTAT-3’ and 5’-CTCCAGCTCTATGTTGACAAA-3’), and cellular GAPDH mRNA (5’-GGTATCGTGGAAGGACTCATGAC-3’ and 5’-ATGCCAGTGAGCTTCCCGTTCAG-3’). Data were analyzed as described previously [83] and levels of GAPDH were used for normalization.

### Analysis of fusion pH

Fusion pH was determined as described previously [84]. In brief, human red blood cell ghosts were incubated with R18-labeled virus of strains PAN, THW, and MAL. Unbound virus was removed and the virus-ghost suspension incubated at different pH values. Finally, the fluorescence dequenching of R18 was recorded and used to determine the EC_50_, which specifies the fusion pH, and the Hill coefficient.

### Mouse experimentation

Female BALB/c mice (Charles River, Sulzfeld, Germany) were housed under pathogen-free conditions in biosafety level 2, according to the German Animal Protection Law (Tierschutzgesetz TierSchG). The animal experiments were approved by the local authorities (Landesamt für Gesundheit und Soziales Berlin LAGeSo, Reg No.: G0206/12). Eight-week-old female mice orally received regorafenib (100 mg/kg, a dose shown to be not toxic for mice [15]) and vehicle control, respectively, formulated as solution in PEG400/125 mM aqueous methanesulfonic acid (80/20) with seven mice per group. Four h later, mice were intranasally infected with 4.4 x 10^4^ PFU of IAV strain A/England/195/2009(H1N1). Twenty-four h after the first dose, regorafenib and vehicle, respectively, were administered again. The mice were euthanized 48 h p.i. and the viral load in the lungs was determined. To this end, the lungs were homogenized, cellular debris was removed by centrifugation, and the amount of infectious virus progeny was determined by plaque assay as described above.

### Resistance assay

MDCK cells were washed with PBS and pre-incubated for 2 h with the different chemical compounds at the indicated concentrations or an equivalent of DMSO under normal culture conditions. Then, cells were inoculated with an MOI of 0.001 of IAV strain A/England/195/2009–M2_N31S_ or WSN for 30 min at 4°C. Unbound virus was removed and the cells cultivated for 24 h in the presence of small molecules and TPCK-treated trypsin. Tissue culture supernatants were harvested, cells and debris removed by centrifugation (5 min, 4°C, 1,400 x g), and the supernatants stored at 4°C. Virus load in cleared supernatants was quantified by plaque assay. This procedure was performed five (WSN) or eight (A/England/195/2009–M2_N31S_) times, always using the supernatant of the latest passage for inoculation.

## Supporting information

S1 Fig Graphical representation of strain-independent genes and processes. The top ten ranking clusters according to DAVID functional annotation clustering (Fig 1D) (light blue) and the top ten ranking canonical pathways according to IPA (Fig 1E) (dark blue), the corresponding strain-independent genes (red) and the higher order cellular processes (green) have been illustrated as interaction map.
(PDF)

S2 Fig Strain-specifically required genes can be assigned to one of four clusters. (A) Strain-specific genes were identified by mixed effects analysis and clustered using the CLICK algorithm [79]. Data represent mean and standard deviation (SD) of the normalized viral load upon knockdown of the genes in the individual clusters. A.u.: arbitrary units. Twenty-one genes (B) could not be assigned to any cluster. Data represent the mean of the normalized viral load for the siRNAs targeting the individual genes. Data analyzed are from the screen outlined in Fig 1A.
(PDF)

S3 Fig Several gene categories are strain-specifically required. Strain-specific gene categories were identified by mixed effects analysis. Exemplary gene categories are shown. Data represent average virus titers upon knockdown of genes of the respective categories relative to negative control. Number of tested genes and siRNAs associated with the respective category as well as p-value of mixed effects analysis are specified in boxes. (A) Gene Ontology (GO) category nucleotide-binding domain, leucine rich repeat containing receptor signaling pathway. This pathway activates NF-кB [85]. (B) GO category regulation of RNA splicing. (C) GO category RNA polymerase II transcription cofactor activity.
(PDF)

S4 Fig CRISPR/Cas9-mediated knockout effects on viral replication for target genes of regorafenib/sorafenib. A549-CRISPR/Cas9 cells were infected with WSN for 36 h. Virus load was assessed by fluorescent focus assay. Genes selected are major targets of regorafenib/sorafenib [15, 16, 23]. Data represent average virus titers ± SEM of technical replicates (n = 3).
(PDF)

S5 Fig UBKIs do not affect internalization of CME cargos. (A) A549 cells were serum-starved for 3 h and subsequently pre-treated with small molecules (dynasore: 100 μM, regorafenib/sorafenib: 3 μM) or an equivalent amount of DMSO for 30 min. Cells were incubated at 4°C with Alexa Fluor 647-labeled epidermal growth factor (EGF) for 1 h. To induce internalization of EGF, cells were incubated at 37°C for 10 min. The amount of internalized EGF was quantified by flow cytometry. Data represent mean ± SEM of n = 3 independent experiments specified in arbitrary units (a.u). The one-way ANOVA of the log-transformed data provided evidence for different mean values (p = 0.052). Unadjusted post-tests led to a significant difference between DMSO and dynasore (p = 0.024). The adjusted p-value for comparison with DMSO was 0.071 for dynasore and non-significant (ns) for regorafenib and sorafenib. (B) Cells treated as in (A) but using Alexa Fluor 488-labeled transferrin. One-way ANOVA of the log-transformed data suggests significantly different mean values (p = 0.028). In contrast to regorafenib and sorafenib, adjusted post-tests for multiple testing led to a significant difference between DMSO and dynasore (p = 0.037).
(PDF)

S6 Fig UBKIs impair post-internalization processing of CME cargos. (A) A549 cells were pre-treated with small molecules or DMSO as described for Fig 4 before incubation at 4°C with EGF-A647. After a 10 min pulse, cells were further incubated at 37°C for 30, 60, or 120 min with EGF-free medium before fixation. The amount of internalized EGF-A647 was quantified by flow cytometry. Data represent mean (n = 3) ± SEM of independent experiments relative to obtained values after 10 min. (B) Same experimental setup as in (A) but using transferrin-Alexa-488. Two-way ANOVA for (A) and (B) suggests that time and group are significant factors, whereas the interaction is not significant. Comparison with the DMSO control at the respective time point was adjusted for multiple testing: *: p-value ≤ 0.05, **: p-value ≤ 0.01.
(PDF)

S7 Fig UBKIs impair vRNP nuclear import. Data were acquired as described in the legend of Fig 5F. Representative micrographs of the x-y plane (large) and the z-axis (narrow) of individual cells are shown. The horizontal z-stacks are identical to those shown in Fig 5F.
(PDF)

S8 Fig Fusion pH of representative IV strains. (A) Virus of strains PAN, THW, and MAL were labeled with the lipophilic dye R18. Labeled viruses were incubated with human red blood cell ghosts followed by incubation at different pH values. Finally, fluorescence dequenching (FDQ) of R18 was recorded. A.u.: arbitrary units (B) The EC_50_ (which defines the fusion pH) and the Hill coefficient of the curves depicted in (A) are shown. EC_50_: pH at which FDQ is half maxima. SEM of EC_50_ and Hill coefficient, respectively, are standard errors determined by nonlinear regression.
(PDF)

S9 Fig Cell viability dose-response curves in different cell types. Cells were cultivated for 48 h in presence of small molecules at different concentrations prior to conduction of WST-1 assay. Data represent signal in WST-1 assay relative to the vehicle control expressed as mean ± SEM of n = 3 technical replicates. (A) HEL cell-derived megakaryocytes. (B) hAECB. (C) MDCK cells. (D) To test for potential cytotoxicity of FLT4 inhibitors at the concentration used in experiments shown in Fig 6, both inhibitors were added to cultures for 48 h at a concentration of 6.25 μM. Data represent mean ± SEM of n = 3 independent experiments (mean values are not significantly different: one-way ANOVA: p = 0.697).
(PDF)

S10 Fig Antiviral efficacy of UBKIs in murine cells and mice. (A and B) Dose-response curves in MLE 12 cells. For determination of virus replication, MLE 12 cells were pre-treated with regorafenib (A) or sorafenib (B) at different concentrations for 2 h, infected with IV strain WSN and cultivated for 36 h in presence of small molecules. Virus titers in tissue culture supernatants were determined as described in ‘Materials and Methods’. Data represent mean ± SEM of n = 3 technical replicates. For determination of cell viability, MLE 12 cells were cultivated for 36 h in the presence of small molecules at different concentrations prior to WST-1 assay. Data indicate signal intensity in WST-1 assay relative to vehicle control and represent mean (n = 2) ± SEM. (C) Eight-week-old female mice (n = 7) were orally treated with regorafenib and vehicle control, respectively, with seven mice per group. Four h later, mice were intranasally infected with 4.4 x 10^4^ PFU of IAV strain A/England/195/2009(H1N1). Twenty-four h after the first dose, regorafenib and vehicle, respectively, were administered again. The mice were euthanized 48 h p.i. and the viral load in the lung homogenates determined. Individual values, mean and SEM are presented. The virus load of treated animals was not significantly different to controls (p = 0.317, Wilcoxon rank-sum (Mann-Whitney) test).
(PDF)

S11 Fig UBKIs impose a barrier against resistance development. MDCK cells were pre-treated with 3 μM regorafenib or an equivalent amount of DMSO for 2 h, infected with MOI 0.001 of IV strain WSN and cultivated for 24 h in presence of reagents. Virus titer in tissue culture supernatants was determined and the supernatants were used for inoculation of the next passage. The experiment was terminated after 5 passages. Data represent mean ± SD of technical replicates.
(PDF)

S12 Fig Missing correlation of IFN induction with virus replication. Based on the siRNA level data of the WSN screen the IFN induction data were plotted vs. the virus replication data. Since no correlation of signals in the IFN induction assay and the virus replication assay was observed, all sample siRNAs were treated as non-inducers of IFN.
(PDF)

S1 Table Composition of the siRNA library. (DOCX)

S2 Table Result of virus replication siRNA screen on siRNA level. (XLSX)

S3 Table Result of virus replication siRNA screen on gene level. (XLSX)

S4 Table Gene enrichment analysis with DAVID. (XLSX)

S5 Table Gene enrichment analysis with IPA. (XLSX)

S6 Table Differentially required gene categories associated with NF-kB activation. (XLSX)

S7 Table Differentially required gene categories associated with regulation of RNA splicing. (XLSX)

S8 Table Differentially required gene categories associated with cellular RNA polymerase activity. (XLSX)

S9 Table Genes significantly different required between the four virus strains. (XLSX)

S10 Table Gene groups significantly different required between the four virus strains. (XLSX)

S11 Table Result of small molecule screen. (DOCX)

S12 Table GuideRNA sequences for CRISPR/Cas9-mediated gene deletion. (XLSX)
