# Supplementary figures and images for: RNAi-based small molecule repositioning reveals clinically approved urea-based kinase inhibitors as broadly active antivirals
Source: PLoS Pathog. 2019 Mar 18;15(3):e1007601. doi: 10.1371/journal.ppat.1007601 (PMC6422253; doi:10.1371/journal.ppat.1007601)

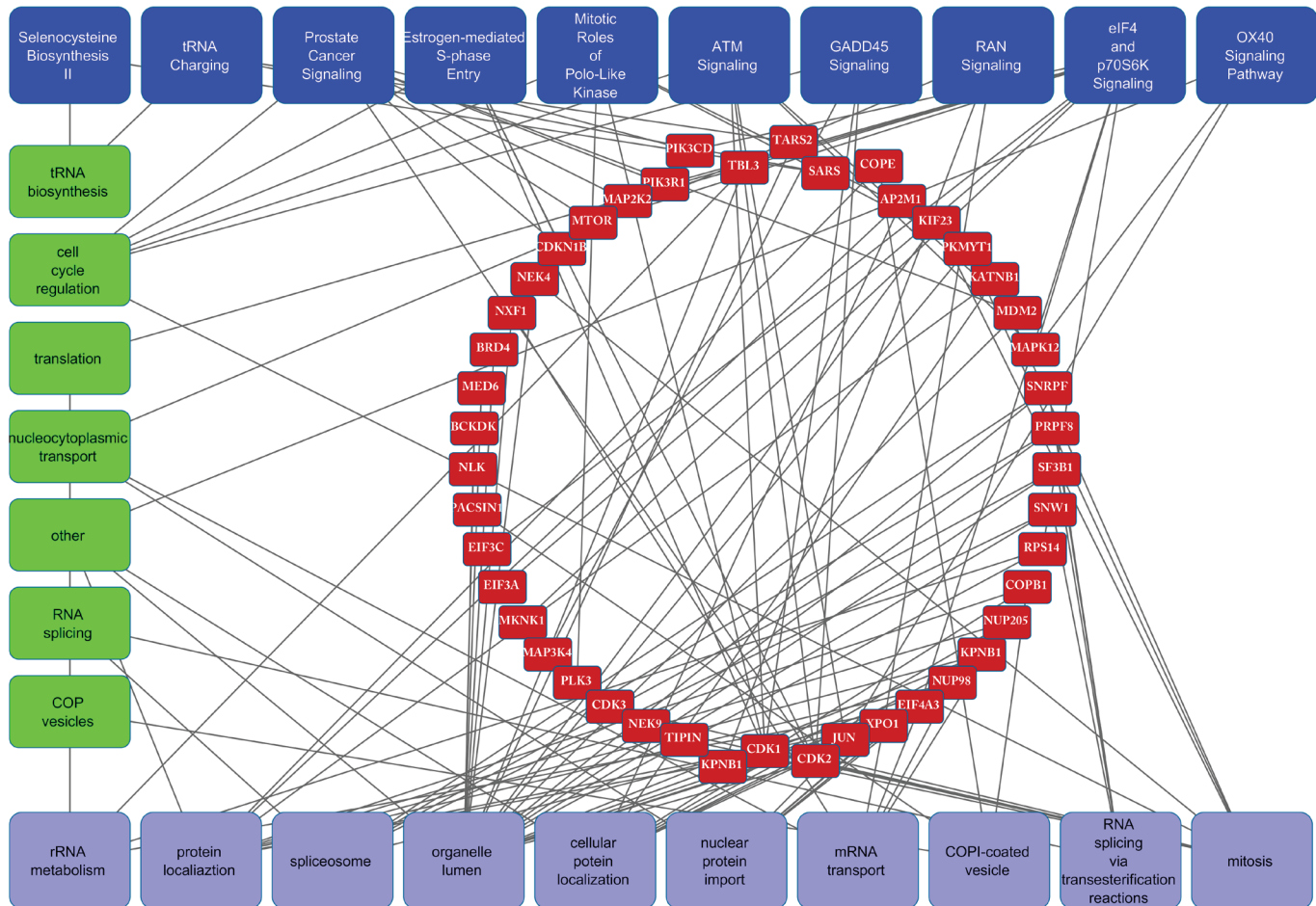

Supplement: S1 Fig — The top ten ranking clusters according to DAVID functional annotation clustering (Fig 1D) (light blue) and the top ten ranking canonical pathways according to IPA (Fig 1E) (dark blue), the corresponding strain-independent genes (red) and the higher order cellular processes (green) have been illustrated as interaction map. (PDF) [file ppat.1007601.s001.pdf]

**A**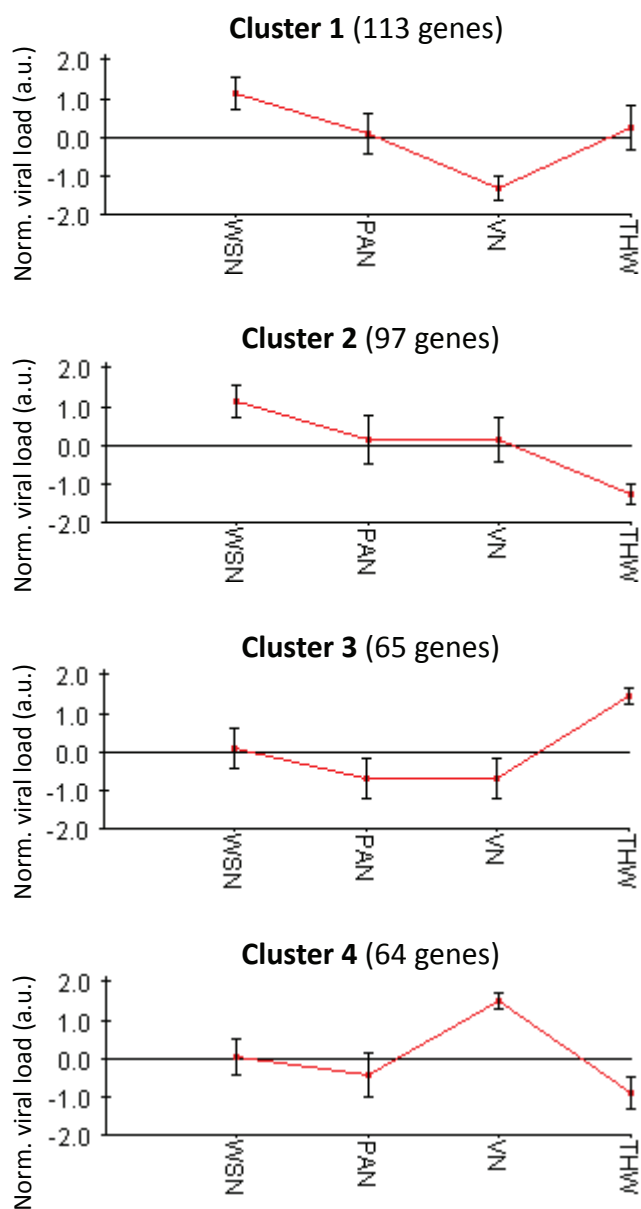**B**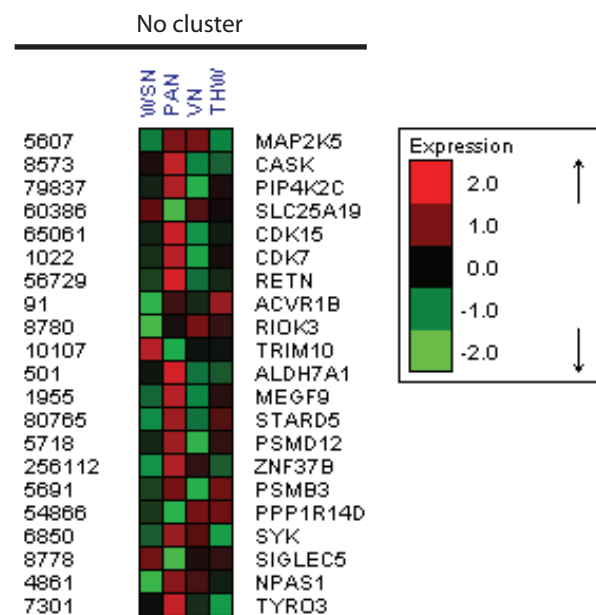

Supplement: S2 Fig — (A) Strain-specific genes were identified by mixed effects analysis and clustered using the CLICK algorithm [79]. Data represent mean and standard deviation (SD) of the normalized viral load upon knockdown of the genes in the individual clusters. A.u.: arbitrary units. Twenty-one genes (B) could not be assigned to any cluster. Data represent the mean of the normalized viral load for the siRNAs targeting the individual genes. Data analyzed are from the screen outlined in Fig 1A. (PDF) [file ppat.1007601.s002.pdf]

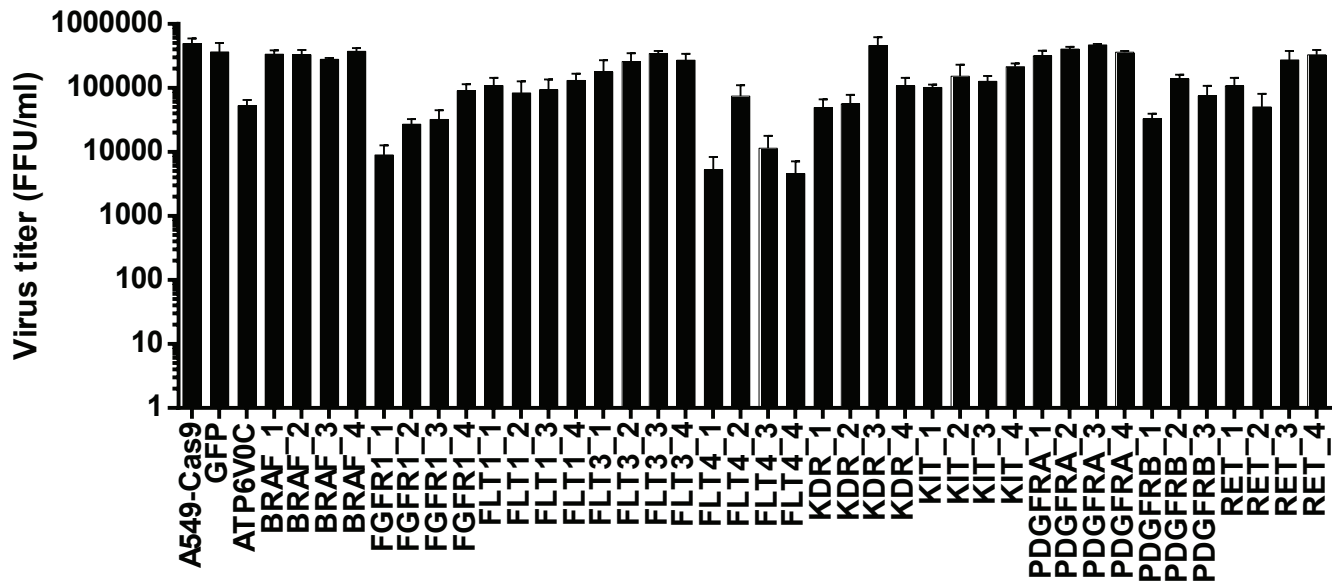

Supplement: S4 Fig — A549-CRISPR/Cas9 cells were infected with WSN for 36 h. Virus load was assessed by fluorescent focus assay. Genes selected are major targets of regorafenib/sorafenib [15, 16, 23]. Data represent average virus titers ± SEM of technical replicates (n = 3). (PDF) [file ppat.1007601.s004.pdf]

**A**

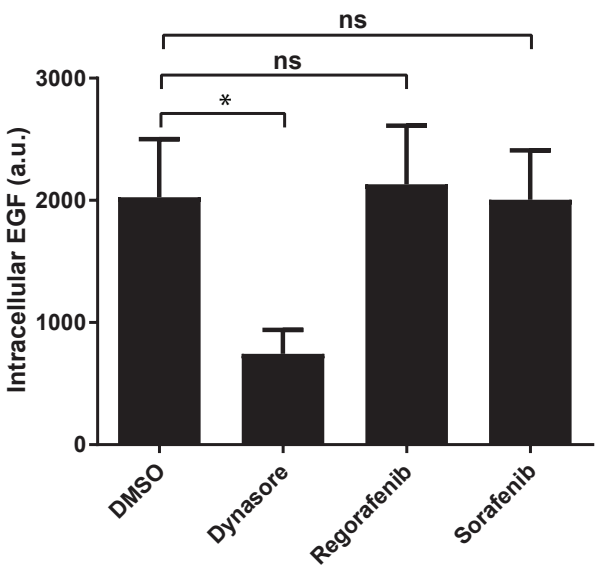

**B**

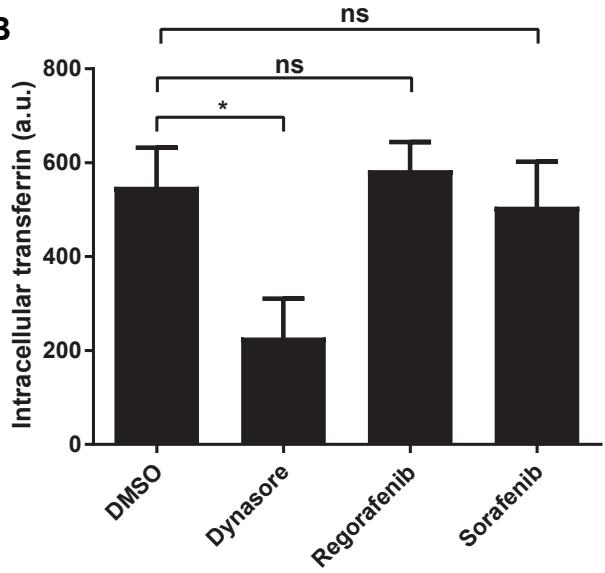

Supplement: S5 Fig — (A) A549 cells were serum-starved for 3 h and subsequently pre-treated with small molecules (dynasore: 100 μM, regorafenib/sorafenib: 3 μM) or an equivalent amount of DMSO for 30 min. Cells were incubated at 4°C with Alexa Fluor 647-labeled epidermal growth factor (EGF) for 1 h. To induce internalization of EGF, cells were incubated at 37°C for 10 min. The amount of internalized EGF was quantified by flow cytometry. Data represent mean ± SEM of n = 3 independent experiments specified in arbitrary units (a.u). The one-way ANOVA of the log-transformed data provided evidence for different mean values (p = 0.052). Unadjusted post-tests led to a significant difference between DMSO and dynasore (p = 0.024). The adjusted p-value for comparison with DMSO was 0.071 for dynasore and non-significant (ns) for regorafenib and sorafenib. (B) Cells treated as in (A) but using Alexa Fluor 488-labeled transferrin. One-way ANOVA of the log-transformed data suggests significantly different mean values (p = 0.028). In contrast to regorafenib and sorafenib, adjusted post-tests for multiple testing led to a significant difference between DMSO and dynasore (p = 0.037). (PDF) [file ppat.1007601.s005.pdf]

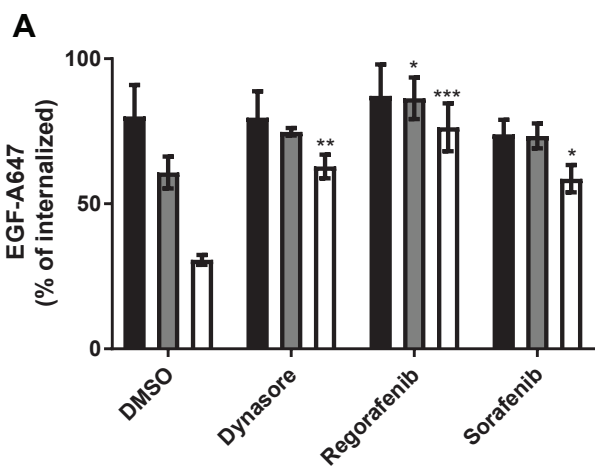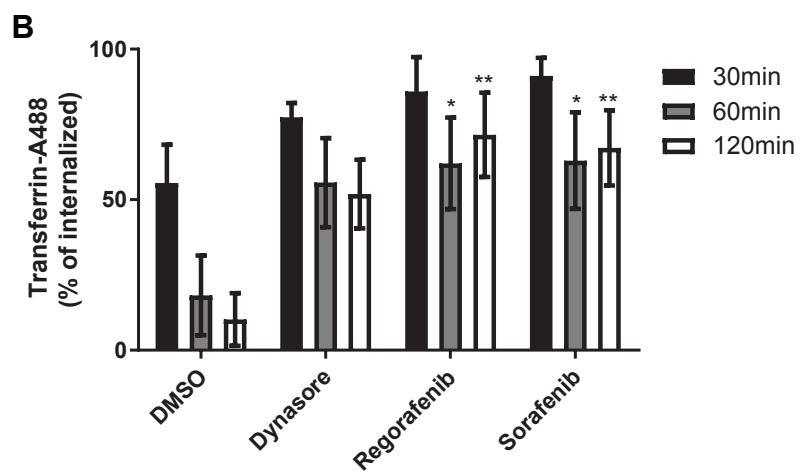

Supplement: S6 Fig — (A) A549 cells were pre-treated with small molecules or DMSO as described for Fig 4 before incubation at 4°C with EGF-A647. After a 10 min pulse, cells were further incubated at 37°C for 30, 60, or 120 min with EGF-free medium before fixation. The amount of internalized EGF-A647 was quantified by flow cytometry. Data represent mean (n = 3) ± SEM of independent experiments relative to obtained values after 10 min. (B) Same experimental setup as in (A) but using transferrin-Alexa-488. Two-way ANOVA for (A) and (B) suggests that time and group are significant factors, whereas the interaction is not significant. Comparison with the DMSO control at the respective time point was adjusted for multiple testing: *: p-value ≤ 0.05, **: p-value ≤ 0.01. (PDF) [file ppat.1007601.s006.pdf]

DMSO

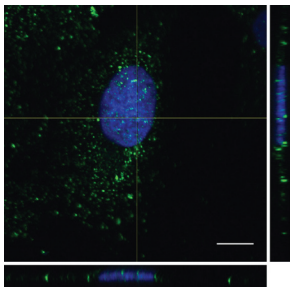

Importazole

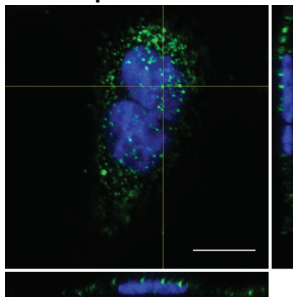

Regorafenib

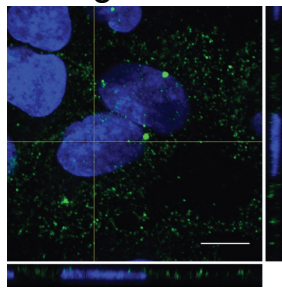

Sorafenib

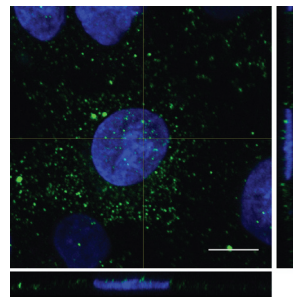

Supplement: S7 Fig — Data were acquired as described in the legend of Fig 5F. Representative micrographs of the x-y plane (large) and the z-axis (narrow) of individual cells are shown. The horizontal z-stacks are identical to those shown in Fig 5F. (PDF) [file ppat.1007601.s007.pdf]

**A**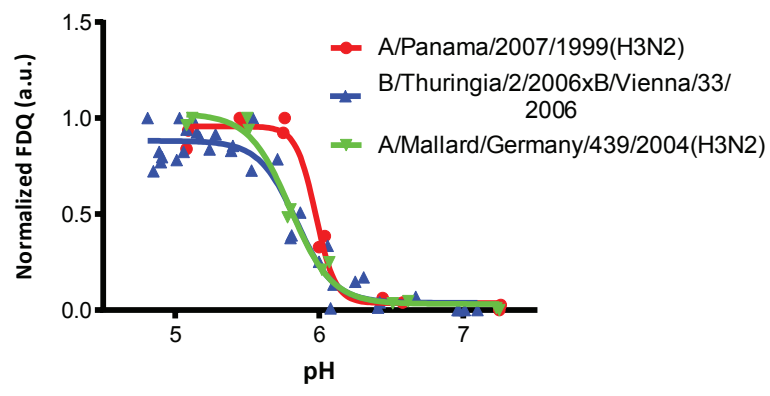**B**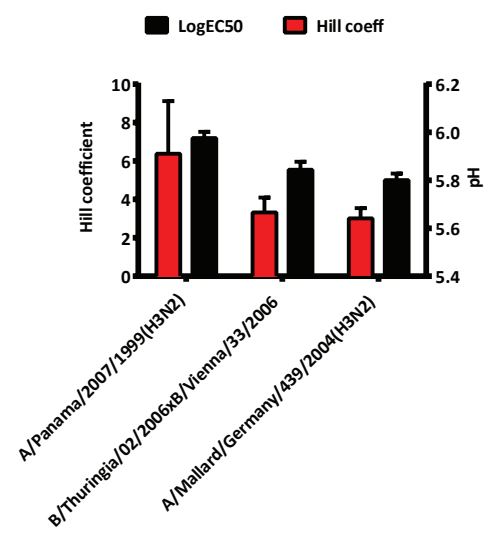

Supplement: S8 Fig — (A) Virus of strains PAN, THW, and MAL were labeled with the lipophilic dye R18. Labeled viruses were incubated with human red blood cell ghosts followed by incubation at different pH values. Finally, fluorescence dequenching (FDQ) of R18 was recorded. A.u.: arbitrary units (B) The EC50 (which defines the fusion pH) and the Hill coefficient of the curves depicted in (A) are shown. EC50: pH at which FDQ is half maxima. SEM of EC50 and Hill coefficient, respectively, are standard errors determined by nonlinear regression. (PDF) [file ppat.1007601.s008.pdf]

**A****HEL-derived megakaryocytes**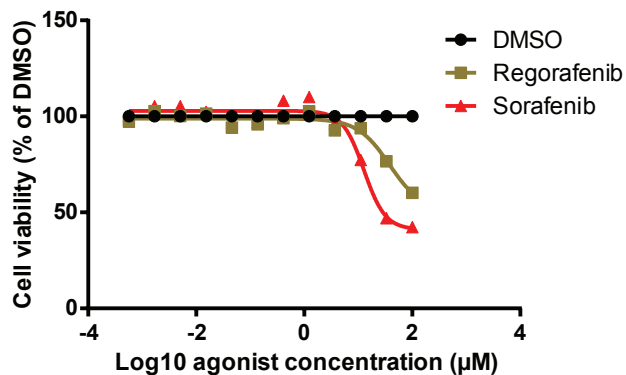**B****hAECB**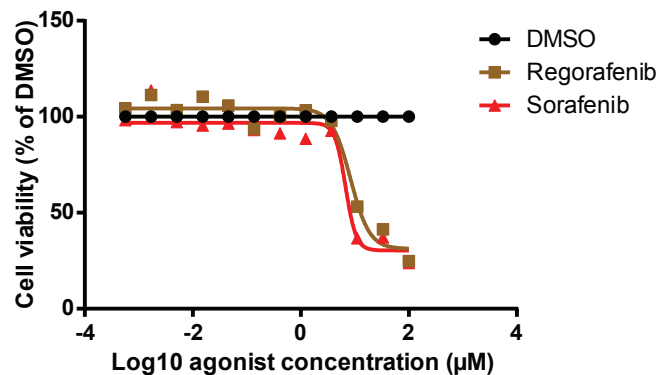**C****MDCK**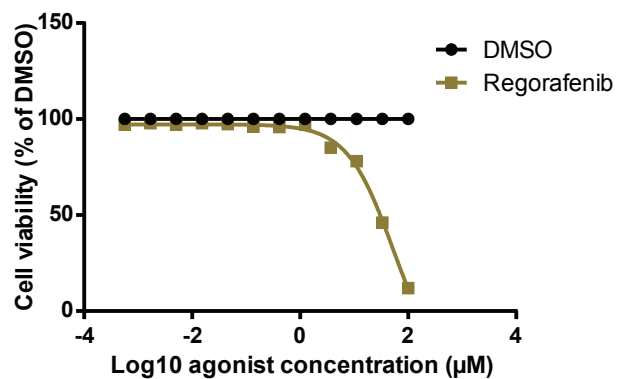**D****hAECB**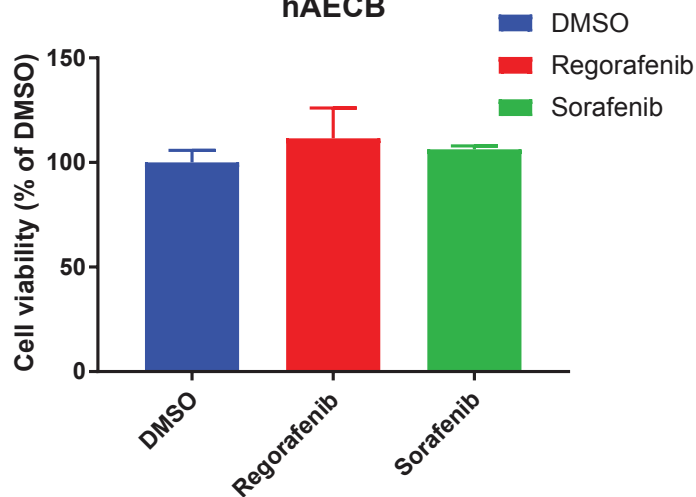

Supplement: S9 Fig — Cells were cultivated for 48 h in presence of small molecules at different concentrations prior to conduction of WST-1 assay. Data represent signal in WST-1 assay relative to the vehicle control expressed as mean ± SEM of n = 3 technical replicates. (A) HEL cell-derived megakaryocytes. (B) hAECB. (C) MDCK cells. (D) To test for potential cytotoxicity of FLT4 inhibitors at the concentration used in experiments shown in Fig 6, both inhibitors were added to cultures for 48 h at a concentration of 6.25 μM. Data represent mean ± SEM of n = 3 independent experiments (mean values are not significantly different: one-way ANOVA: p = 0.697). (PDF) [file ppat.1007601.s009.pdf]

**A**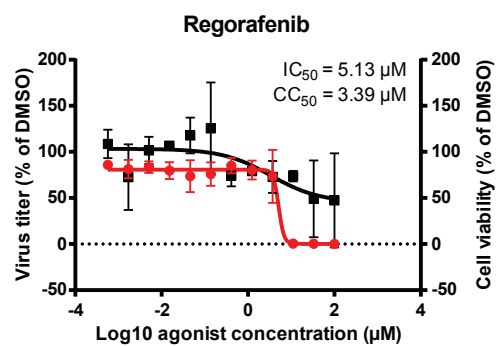**B**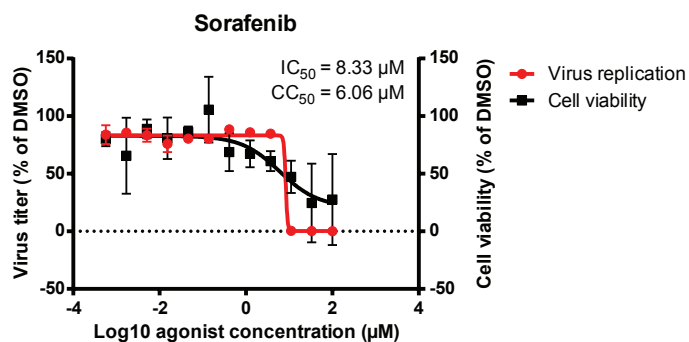**C**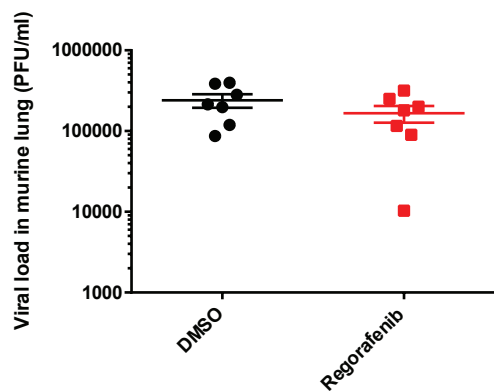

Supplement: S10 Fig — (A and B) Dose-response curves in MLE 12 cells. For determination of virus replication, MLE 12 cells were pre-treated with regorafenib (A) or sorafenib (B) at different concentrations for 2 h, infected with IV strain WSN and cultivated for 36 h in presence of small molecules. Virus titers in tissue culture supernatants were determined as described in ‘Materials and Methods’. Data represent mean ± SEM of n = 3 technical replicates. For determination of cell viability, MLE 12 cells were cultivated for 36 h in the presence of small molecules at different concentrations prior to WST-1 assay. Data indicate signal intensity in WST-1 assay relative to vehicle control and represent mean (n = 2) ± SEM. (C) Eight-week-old female mice (n = 7) were orally treated with regorafenib and vehicle control, respectively, with seven mice per group. Four h later, mice were intranasally infected with 4.4 x 10^4^ PFU of IAV strain A/England/195/2009(H1N1). Twenty-four h after the first dose, regorafenib and vehicle, respectively, were administered again. The mice were euthanized 48 h p.i. and the viral load in the lung homogenates determined. Individual values, mean and SEM are presented. The virus load of treated animals was not significantly different to controls (p = 0.317, Wilcoxon rank-sum (Mann-Whitney) test). (PDF) [file ppat.1007601.s010.pdf]

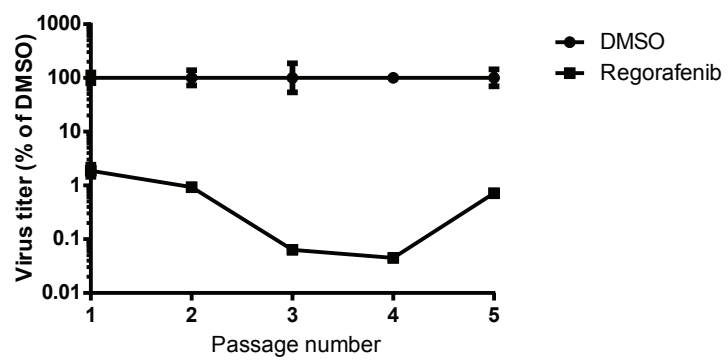

Supplement: S11 Fig — MDCK cells were pre-treated with 3 μM regorafenib or an equivalent amount of DMSO for 2 h, infected with MOI 0.001 of IV strain WSN and cultivated for 24 h in presence of reagents. Virus titer in tissue culture supernatants was determined and the supernatants were used for inoculation of the next passage. The experiment was terminated after 5 passages. Data represent mean ± SD of technical replicates. (PDF) [file ppat.1007601.s011.pdf]

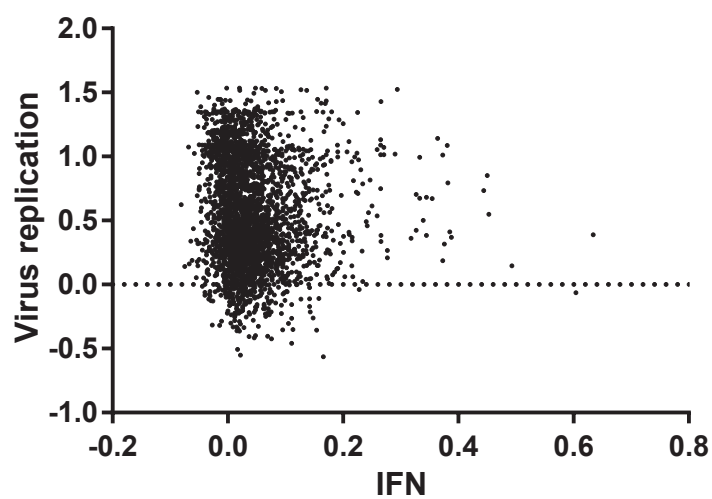

Supplement: S12 Fig — Based on the siRNA level data of the WSN screen the IFN induction data were plotted vs. the virus replication data. Since no correlation of signals in the IFN induction assay and the virus replication assay was observed, all sample siRNAs were treated as non-inducers of IFN. (PDF) [file ppat.1007601.s012.pdf]
