# Supplementary material for: RNAi-based small molecule repositioning reveals clinically approved urea-based kinase inhibitors as broadly active antivirals
Source: PLoS Pathog. 2019 Mar 18;15(3):e1007601. doi: 10.1371/journal.ppat.1007601 (PMC6422253; doi:10.1371/journal.ppat.1007601)
Supplement: S3 Fig — Strain-specific gene categories were identified by mixed effects analysis. Exemplary gene categories are shown. Data represent average virus titers upon knockdown of genes of the respective categories relative to negative control. Number of tested genes and siRNAs associated with the respective category as well as p-value of mixed effects analysis are specified in boxes. (A) Gene Ontology (GO) category nucleotide-binding domain, leucine rich repeat containing receptor signaling pathway. This pathway activates NF-кB [85]. (B) GO category regulation of RNA splicing. (C) GO category RNA polymerase II transcription cofactor activity. (PDF) [file ppat.1007601.s003.pdf]

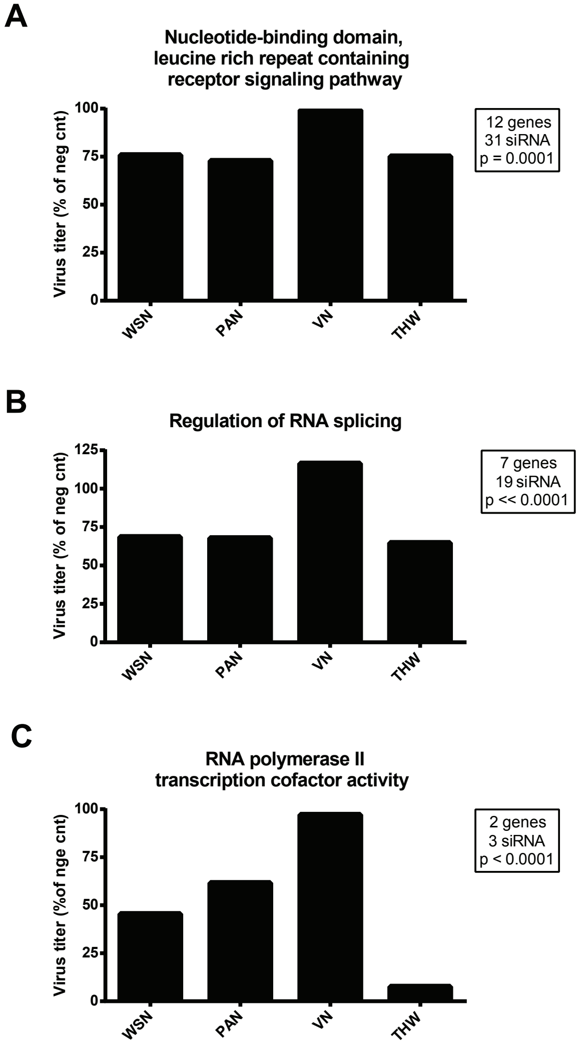
A
Nucleotide-binding domain, leucine rich repeat containing receptor signaling pathway
Virus titer (% of neg cnt)
100
75
50
25
0
WSN
PAN
VN
THW
12 genes
31 siRNA
p = 0.0001
B
Regulation of RNA splicing
Virus titer (% of neg cnt)
125
100
75
50
25
0
WSN
PAN
VN
THW
7 genes
19 siRNA
p << 0.0001
C
RNA polymerase II transcription cofactor activity
Virus titer (%of nge cnt)
100
75
50
25
0
WSN
PAN
VN
THW
2 genes
3 siRNA
p < 0.0001
