# Supplementary material for: RNAi-based small molecule repositioning reveals clinically approved urea-based kinase inhibitors as broadly active antivirals
Source: PLoS Pathog. 2019 Mar 18;15(3):e1007601. doi: 10.1371/journal.ppat.1007601 (PMC6422253; doi:10.1371/journal.ppat.1007601)
Supplement: S1 Table — (DOCX) [file ppat.1007601.s013.docx]

***Supplementary table 1***

| Gene sub-set | Number of genes | Reason to choose sub-set | Reference |
| --- | --- | --- | --- |
| Validation screen of Karlas *et al.* | 461 | Genes identified by our group | {Karlas, 2010 #21} |
| Genes identified in ≥ 2 studies | 128 | Genes confirmed by independent groups | {Watanabe, 2010 #17} |
| Human kinome | 717 | Kinase/signaling most overrepresented gene category in multiple studies | {Shaw, 2011 #22} |
| TOTAL | 1208^§^ | - | - |

^§^ Due to a partial overlap of the gene sub-sets, the total number of genes is smaller than the sum of the genes in the individual sub-sets.
