# Supplementary material for: RNAi-based small molecule repositioning reveals clinically approved urea-based kinase inhibitors as broadly active antivirals
Source: PLoS Pathog. 2019 Mar 18;15(3):e1007601. doi: 10.1371/journal.ppat.1007601 (PMC6422253; doi:10.1371/journal.ppat.1007601)
Supplement: S11 Table — (DOCX) [file ppat.1007601.s023.docx]

**Table S11. Result of small molecule screen**

| Agent | Target gene | IC_50_ [µM] | CC_50_ [µM] | SI |
| --- | --- | --- | --- | --- |
| alvocidib | CDK1, CDK2, CDK3 | 0.10 | 0.48 | 4.74 |
| abiraterone | CYP17A1 | not active | not toxic | not toxic |
| abiraterone acetate | CYP17A1 | 115.82 | not toxic | not toxic |
| ketoconazole | CYP17A1 | 40.30 | not toxic | not toxic |
| vemurafenib | FGR, TNK2 | 4.95 | 355.13 | 71.73 |
| axitinib | FLT4 | 63.12 | not toxic | not toxic |
| cabozantinib | FLT4 | 4.98 | 32.37 | 6.50 |
| CEP 7055 | FLT4 | - | - | - |
| motesanib | FLT4 | 17.56 | not toxic | not toxic |
| pazopanib | FLT4, LCK | 21.07 | not toxic | not toxic |
| pazopanib HCl | FLT4, LCK | 11.68 | not toxic | not toxic |
| regorafenib | FLT4 | 0.89 | 14.15 | 15.93 |
| sorafenib | FLT4 | 0.44 | 36.26 | 81.66 |
| sunitinib | FLT4 | 5.54 | 14.76 | 2.66 |
| tivozanib | FLT4 | 2.83 | not toxic | not toxic |
| vandetanib | FLT4 | 6.01 | 193.51 | 32.18 |
| ruxolitinib | JAK1 | 41.42 | not toxic | not toxic |
| tofacitinib | JAK1 | 62.31 | not toxic | not toxic |
| dasatinib | LCK | 2.42 | 37.59 | 15.53 |
| selumetinib | MAP2K2 | not active | not toxic | not toxic |
| trametinib | MAP2K2 | 90.73 | not toxic | not toxic |
| talmapimod | MAPK12 | 8.31 | not toxic | not toxic |
| everolimus | MTOR | 28.39 | not toxic | not toxic |
| OSI-027 | MTOR | 0.97 | not toxic | not toxic |
| pimecrolimus | MTOR | 0.02 | not toxic | not toxic |
| sirolimus | MTOR | 36.69 | not toxic | not toxic |
| ridaforolimus | MTOR | 51.36 | 69.42 | 1.35 |
| tacrolimus | MTOR | 15.70 | 49.89 | 3.18 |
| temsirolimus | MTOR | 26.10 | 272.07 | 10.42 |
| clopidogrel | P2RY12 | not active | not toxic | not toxic |
| epoprostenol | P2RY12 | not active | not toxic | not toxic |
| ticagrelor | P2RY12 | 32.67 | 183.53 | 5.62 |
| ticlopidine | P2RY12 | not active | not toxic | not toxic |
| prasugrel | P2RY12 | 2.18 | not toxic | not toxic |
| treprostinil | P2RY12 | not active | not toxic | not toxic |
| pictrelisib | PIK3CD | 0.47 | not toxic | not toxic |
| BMS-387032 | PIK3CD | - | - | - |
| buparlisib | PIK3CD | 1.57 | not toxic | not toxic |
| dactolisib | PIK3CD | 95.69 | not toxic | not toxic |
| idelalisib | PIK3CD | not active | not toxic | not toxic |
| PX-866 | PIK3CD | 1.04 | not toxic | not toxic |
| SF 1126 | PIK3CD | 26.67 | 28.77 | 1.08 |
| XL147 | PIK3CD | 8.56 | not toxic | not toxic |

IC_50_, half maximal inhibitory concentration in fluorescent focus assay; CC_50_, half maximal inhibitory concentration in WST-1 assay; SI, selectivity index
